# A pathogen of good taste: genetics of a bacterial host jump of the plant pathogen Xylella fastidiosa from coffee to wine grapes

**DOI:** 10.1099/mgen.0.001447

**Published:** 2025-07-17

**Authors:** Alexandra Katz Kahn, Jasslin Cervantes, Rodrigo P. P. Almeida

**Affiliations:** 1 Department of Environmental Science, Policy and Management, University of California Berkeley, Berkeley, CA 94720, USA

**Keywords:** emerging pathogen, host jump, plant pathogen, *Xylella fastidiosa*, *Coffea arabica*, *Vitis vinifera*

## Abstract

When pathogens are repeatedly introduced into new environments, host jumps may occur into naïve taxa. Given the magnitude of the global plant trade, this process can lead to frequent disease emergence as interactions between previously isolated pathogens and new plant hosts become possible. *Xylella fastidiosa* is a recurring nuisance. This bacterial pathogen has recently emerged in novel geographic locations infecting a breadth of host plants. An introduction of *X. fastidiosa* subsp. *fastidiosa* from Central America to the USA several hundred years ago has since been the source of outbreaks across the globe. In the USA, particularly in California, the introduced bacterium is frequently found in European grapevine (*Vitis vinifera*). In this study, we demonstrated that the introduced strains do not persistently infect *Coffea arabica*. Furthermore, we did not observe an overall increase in the virulence of the introduced strains towards coffee, indicating a lack of hypervirulence. Then, using *X. fastidiosa* subsp. *fastidiosa* whole-genome sequences, 15 from the source region of Costa Rica and 289 from the introduced clade, we tested for traces of adaptation to grapevines. We found both genes and SNPs that are associated with the host shift to grapevines. These results support the hypothesis that a host jump with genetic adaptation occurred following the introduction of the pathogen into the USA.

**Impact Statement**

Just in 2024, the scientific community became aware of novel *Xylella fastidiosa* outbreaks in grapevines in Portugal and Italy. The pathogen continues to emerge, sometimes leading to epidemics, and always leading to uncertain plant health futures. This study investigates an important introduction event, leading to a new disease, revealing evidence of a host jump marked by the pathogen’s diminished ability to infect a common host in its native range. By examining historical introduction events, we provide critical insights into the evolutionary and ecological mechanisms driving pathogen adaptation, informing strategies to anticipate and mitigate the impacts of contemporary outbreaks occurring at an accelerating pace.

## Data Summary

All whole-genome sequences used in this paper are openly available in NCBI, and reference numbers can be found in Table S1 (available in the online Supplementary Material).

## Introduction

Host jumps – where a pathogen adapts to a novel host – can arise from initially suboptimal interactions in a new host environment that improve with evolutionary adaptation [1]. These transitions occur along a continuum and are often driven by molecular mechanisms such as gene gain or loss, recombination, mutations or genomic rearrangement [2]. A novel host encounter, or host shift, may or may not result in pathogen speciation, particularly when phenotypic plasticity allows for some niche generalism [3]. In agriculture, host jumps pose a particular threat because most crops are cultivated in genetically uniform monocultures that facilitate disease spread [4]. California (CA), as the epicentre of the US fruit, vegetable and nut production, and much international plant trade, is a critical region for monitoring phytopathogen emergence and movement [5]. It can act both as a source and a recipient for the global circulation of pathogens, including *Xylella fastidiosa*, a xylem-limited, insect-transmitted bacterium responsible for a wide range of plant diseases.

Phylogenetic and historical data suggest that *X. fastidiosa* subsp. *fastidiosa* originated in Central America and was likely introduced into the USA via live-plant imports such as *Coffea arabica* in the 1700s [6,9]. Since its introduction into the USA, the pathogen has continued to spread, including recent introductions into Europe, the Middle East and Asia [10,13]. Outbreaks of *X. fastidiosa* subsp. *fastidiosa* have been devastating to the grape (*Vitis vinifera*) industry [14]. In contrast, in its presumed region of origin, Central America, *X. fastidiosa* infects *C. arabica* and other hosts with relatively mild symptoms and limited economic impact [7]. In Costa Rica (CR), for example, subsp. *fastidiosa* is known to infect *C. arabica*, *Vinca* spp., guava (*Psidium guajava*) and avocado (*Persea americana*), but disease severity in these hosts is generally low and has not been rigorously quantified [9]. The contrast between high virulence in US grapevines and low impact in CR coffee suggests a possible host jump.

Genetic evidence supports this scenario. A study by Castillo *et al*. [9] found patterns within subsp. *fastidiosa* consistent with a bottleneck and introduction event, including reduced Tajima’s D in the US population suggesting a recent selective sweep (−0.37 CR, −1.71 USA), increased linkage disequilibrium, and phylogenetic divergence from Central American strains [9]. Whilst a few CR isolates have been detected in *Vitis*, these strains were not able to infect grapevines in inoculation experiments, suggesting that pathogenicity towards grape may have evolved after introduction to the USA [7,15, 16]. Within *Vitis*, resistance and susceptibility to Pierce’s Disease (PD) of grape vary. Native North American species like *Vitis arizonica* and *Muscadinia rotundifolia* exhibit high tolerance, whilst *V. vinifera*, the main cultivated species in CA, is highly susceptible [17]. Even within * V. vinifera*, variation in PD susceptibility exists amongst cultivars [18,20]. The intensity of PD in the USA is characterized by leaf scorch, fruit loss, and vine death and sharply contrasts with the largely asymptomatic infections observed in *C. arabica*, supporting a host-specific adaptation hypothesis [14,18].

Upon introduction into the USA, *X. fastidiosa* subsp. *fastidiosa* encountered novel hosts, vectors, climates and agricultural conditions. These pressures may have selected for strains more specialized to local hosts and climate or, alternatively, for generally increased virulence. The heightened impact on *V. vinifera* in the USA may reflect either adaptation to this host, a broader shift towards hypervirulence or both. To address this uncertainty, we investigated both neutral and adaptive changes during the process of naturalization for this pathogen using whole-genome sequences and a plant-infection experiment.

*C. arabica* in CR is a unique host for multiple subspecies of *X. fastidiosa* [21]. It is frequently infected with mild symptoms and was likely the source of a devastating introduction of subsp. *pauca* into Italy [22]. Nonetheless, the *C. arabica*–*X. fastidiosa* pathosystem is generally understudied. Coffee appears broadly susceptible to multiple subspecies – including subsp. *pauca* and *fastidiosa* – although no data yet exist for subsp. *multiplex* infections in coffee. It has been hypothesized that strains of *X. fastidiosa* do not need to undergo many genetic changes in order to infect *C. arabica*, potentially making it a host susceptible to low-cost host jumps [23]. The advent of coffee production in CA, coupled with historic documentation of subsp. *multiplex* in South America, calls for an investigation of whether *C. arabica* can serve as a host to subsp. *multiplex* [24].

Our major hypotheses are as follows:

1. Loss of infectivity in the ancestral host: US strains have lost the ability to infect *C. arabica*, consistent with a host jump [1] and divergence due to selective pressure for infection in *Vitis*. We expect less persistent infections of the introduced strains in inoculated *C. arabica* plants.
2. Alternatively, US strains could retain infectivity towards *C. arabica* but cause more severe disease than ancestral strains, suggesting a general increase in virulence*,* which we can test through documenting disease symptoms, such as stunting or leaf scorch, during inoculation studies.
3. Experimentally inoculated strains from CR have not been pathogenic to *Vitis* [9], so we hypothesize that strains present in CA have undergone genetic changes that are associated with their ability to infect *V. vinifera* in the USA.

## Results

### Coffee is a poor host of the USA-adapted subsp. *fastidiosa* strains and subsp. *multiplex* strain

**Fig. 1.**
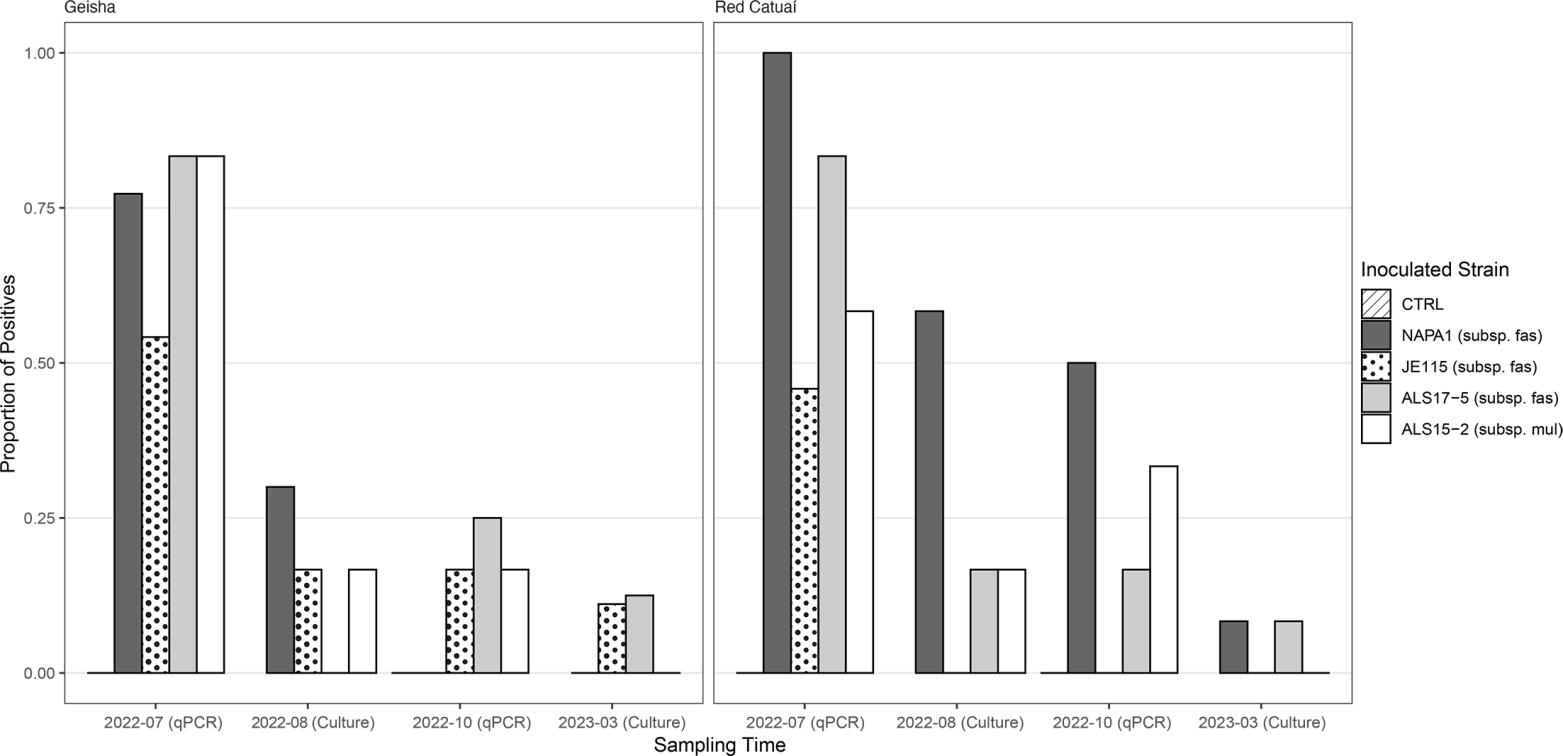
Detection of the strains in *C. arabica* plants based on the proportion of plants testing positive at each detection point. Of the four detection points, two were conducted via qPCR (July 2022 and October 2022), and two were conducted via culturing (August 2022 and March 2023). Data are split between the two *C. arabica* cultivars, Geisha and Red Catuaí, and are all clustered and coloured by inoculation treatment, succinate-citrate-phosphate (SCP) buffer control, one of three strains of subsp. *fastidiosa* or one strain of subsp. *multiplex*. There was no detectable difference in pathogen detection rates between the cultivars Geisha and Red Catuaí; however, there was a significant difference amongst strains used for inoculation.

After inoculating 120 *C. arabica* plants in March 2022, we monitored the persistence of infection over the course of a year. Although infections did not persist in all *C. arabica* plants, we detected bacteria of all four strains by cell culture at two leaf nodes above the initial inoculation point (Fig. 1). Four months after inoculation, in July, most inoculated plants were positive. However, by the final detection 1 year after inoculation, there were only a few persistent infections. There was no detectable difference in pathogen detection rates between the two cultivars, Geisha and Red Catuaí (variety effect *P*=0.76, df=1, chi^2^=0.095). However, there was a significant difference between strains used for inoculation (excluding the control in which no samples tested positive) according to a generalized mixed model (strain effect *P*=0.019, df=3, chi^2^=9.91). Estimates of detection in the model were lowest for the strain from subsp. *multiplex*, showing fewer overall positive samples than from plants infected by subsp. *fastidiosa*. Both *Prunus dulcis* (almond) and *V. vinifera*-derived strains, as well as subsp. *fastidiosa* and subsp. *multiplex*, were able to infect the *C. arabica* plants, but all infections reduced substantially throughout the year. Positive controls [*Helianthus annuus* (sunflower) and *V. vinifera* inoculations] showed that all four bacterial strains were viable and able to infect hosts (Fig. S1).

### No evidence of hypervirulence in coffee plants

**Fig. 2.**
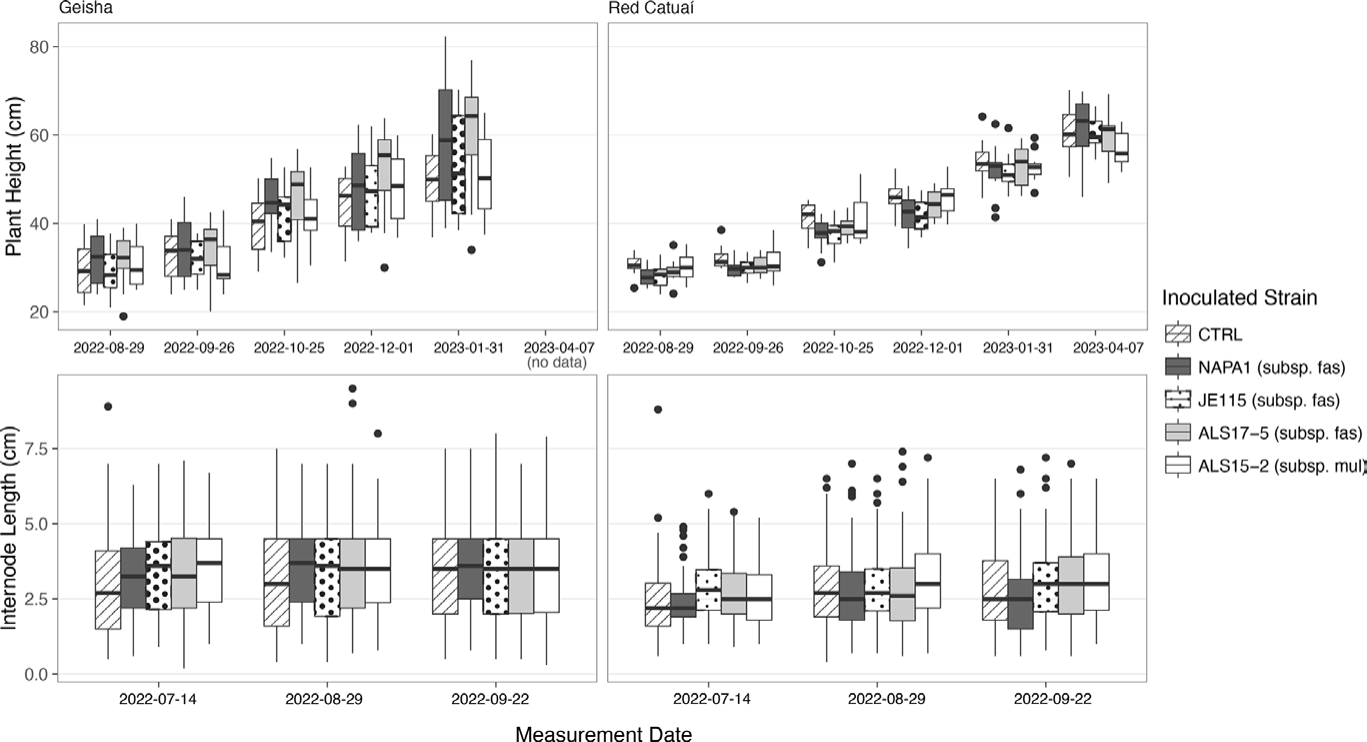
Symptom assessment of *C. arabica* plants cv. Geisha and Red Catuaí as determined by total height and internode length. Data are clustered and coloured by inoculation treatment, SCP buffer control, one of three strains of subsp. *fastidiosa* or one strain of subsp. *multiplex*. There was no difference between the treatment groups for internode length. However, Red Catuaí showed evidence of symptoms associated with the infection – significant stunting of the infected plants compared to the controls.

During the summer of 2022, internode lengths along the main stem were measured for each plant to check for the classic symptom of *X. fastidiosa* in *C. arabica* plants, internode shortening. When tested with a linear mixed model, there was no difference found between the treatment groups for internode length. Heights were also measured from August 2022 to April 2023 in order to detect stunting. Of the two *C. arabica* cultivars, only Red Catuaí showed evidence of symptoms associated with the infection: stunting of the infected plants compared to the controls. Both the interaction between infection status and sampling time was significant, (*P*=3.51e-06, chi^2^=33.2, df=5), and infection status alone was also significant (*P*=0.00223, chi^2^=9.35, df=1) in Red Catuaí in terms of their effects on height. The cultivar Geisha, which exhibited more variation in height, showed no detectable effect of infection status on stunting (*P*=0.23, chi^2^=1.44, df=1). These results can be visualized in Figs2  3.

**Fig. 3.**
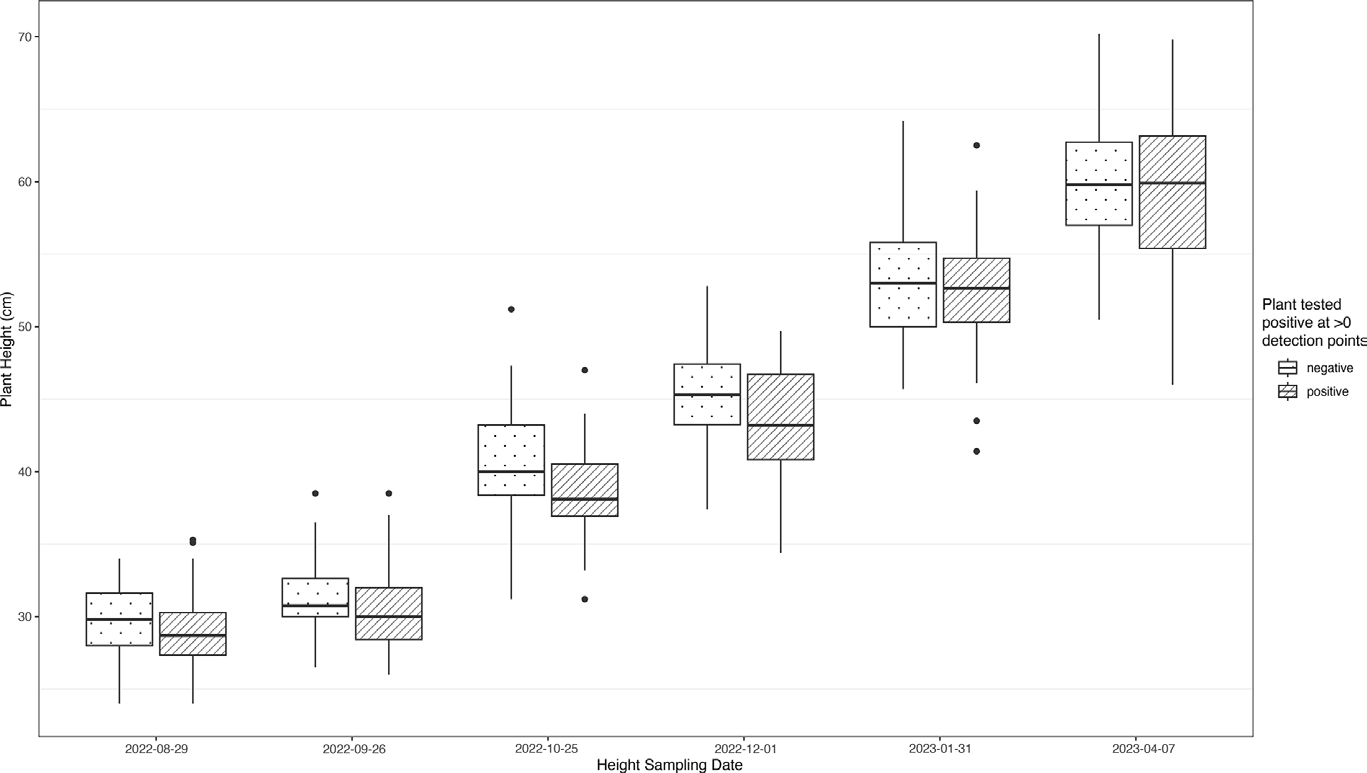
Visualization of plant heights of cultivar Red Catuaí over the course of the experiment. Data are divided into two binary groups, representing whether or not each individual plant tested positive at any time over the course of the experiment. Both the interaction between infection status and sampling time and infection status alone were significant in Red Catuaí. The treatment, comprising three subsp. *fastidiosa* inoculation strains and the SCP buffer negative control, was also highly significant. The cultivar Geisha, which exhibited more variation in height, showed no detectable effect of infection status on stunting.

### Phylogenetic relationships as expected for subsp. *fastidiosa*

The core genome, defined as genes present in ≥95% of strains, contained 2020 genes across all strains, as identified using Panaroo. The full pangenome consisted of 3,500 genes. When analysing only strains derived from *Vitis* hosts, the core genome increased to 2,095 genes, with a smaller pangenome size of 2,756 genes. A core genome maximum-likelihood phylogeny with recombinant regions removed was constructed for all strains, and a simplified cladogram is presented to facilitate visualization of taxonomic relationships. Major clades received strong bootstrap support and reflected expected patterns based on previous studies. Notably, CR strains formed the outgroup to the introduced lineage, which diverged into two primary clades: one predominantly found on the US East Coast (including an outbreak in Taiwan) and another primarily in CA (including an outbreak in Spain) [6].

### Genome-wide association study shows SNPs and genes associated with the host, with moderate specificity

A total of 10,390 SNPs were detected as being significantly associated with the host *Vitis* using the Bonferroni correction for multiple hypothesis testing with *P* values <0.05 using the program Scoary. However, this still does not exclude the possibility of significance via phylogenetic proximity across the tree. Given the few clades of *Vitis*-associated bacteria, this dataset did not offer the power to apply the most conservative method used by Scoary, the worst pairwise comparison *P* value, which would identify only SNPs that have arisen independently across the phylogeny. However, data presented here (Tables 1 and S3) show genes that have a significant corrected *P* value and also have a best pairwise comparison *P*<0.05, showing some indication of independent emergence. Eleven SNPs fit those criteria. Amongst those 11 genes identified with significant SNPs, 3 are only annotated to the level of hypothetical protein. The eight non-hypothetical significant genes are *envC* (murein hydrolase activator EnvC), *fumC* (fumarate hydratase class II), *gcvP* [glycine dehydrogenase (decarboxylating)], *xthA* (exodeoxyribonuclease III), *glnG* (DNA-binding transcriptional regulator NtrC), *uppP* (undecaprenyl-diphosphatase), *yceI_3* (protein YceI) and *aceE* (pyruvate dehydrogenase E1 component). Two identified SNPs were present at lower frequencies in strains from *Vitis* than other hosts, and nine identified SNPs were present at higher frequencies in strains from *Vitis* than other hosts. The mean difference in frequency of these SNPs from *Vitis* and non-*Vitis*-derived strains was 0.38, but none of these SNPs are universally present or absent in either group.

**Table 1.** Genes under positive selection were identified using HyPhy’s Single-Likelihood Ancestor Counting (SLAC) method. Results are based on only samples isolated from *Vitis* spp. The full list of genes that were identified can be found in Table S2. Whole-gene gain and loss genome-wide association study (GWAS) results from Scoary are presented, including loci with best pairwise comparison *P*≤0.125 and Bonferroni *P*<0.05. Annotations and a full list of genes are shown in Table S3. SNP GWAS results from Scoary are presented, including loci with best pairwise comparison *P*<0.05 and Bonferroni-corrected *P*<0.05. Twenty-four SNPs met these criteria, from 11 unique genes. Annotations and a full list of genes are shown in Table S4. treeWAS SNPs are reported fully in the results section of this paper

Method	Genes under positive selection	GWAS SNPs	GWAS genes	treeWAS SNPs
Hypothetical proteins	33	3	32	2
Named genes	38	8	4	3
Mean difference in frequency between populations	–	0.38	0.33	0.63

Gene gain and loss was also tested using Scoary for host associations and the same *P* value corrections to go from 473 genes with Bonferroni *P*<0.05 (data not shown) to 37 genes that also had a best pairwise comparison *P*≤0.125 (Tables 1 and S4). Out of the 37 identified genes, 33 were annotated as hypothetical proteins. The other four loci are *xerC_1/2* (tyrosine recombinase XerC), *hcaB* (3-phenylpropionate-dihydrodiol/cinnamic acid-dihydrodiol dehydrogenase), *mdtA_1/2/3* (multidrug resistance protein MdtA) and a cluster identified using Panaroo that includes the three genes: *cnrA* (nickel and cobalt resistance protein CnrA), *swrC_2* (swarming motility protein SwrC) and *acrF_2* (multidrug export protein AcrF). These genes each belong to a different KEGG orthology (genetic information processing, metabolism, environmental information processing and signalling and cellular processes, respectively). The difference in frequency between the genes in the two populations ranged from 0.19 to 0.52, with no genes fixed as present in one population and absent in another. Most of these genes were present at higher frequencies in the *Vitis* strains than in the non-*Vitis* strains.

Six SNPs in five genes were identified as significantly associated with the host *Vitis* using treeWAS, all with significant subsequent scores: *wbbL* (*P*=0.00E+00), *pilT*_2 (*P*=2.73E-06), *pilT_1* (*P*=0.00E+00), group_1468 (*P*=2.73E-06) and two SNPs in group_86 (*P*=0.00E+00, *P*=0.00E+00) (Fig. S3). The variation between sequences in these genes was further investigated. The SNP identified in the gene *wbbL* as significant by treeWAS was synonymous. The gene *pilT_2* is much more varied across the dataset, with more groups on the nucleotide-based phylogeny, as well as more diversity in amino acid sequences. The change that was identified as significant by treeWAS was synonymous. The genes *pilT_1* and group_1468 are adjacent on the chromosome, implying that both of them being significant could be due to linkage. The SNP identified on *pilT_1* was synonymous; however, the SNP on group_1468 was non-synonymous. The CR strains mostly had a leucine where all other strains had changed to a valine. The first group_86 SNP was a synonymous change from guanine to thymine in the third site of leucine; however, the second SNP was non-synonymous. The two are likely linked. For the non-synonymous substitution, CR and *Prunus* spp. samples had a proline at the site, whilst *Vitis* spp. samples had a serine. No significant genes were found when we ran treeWAS on geographic divisions instead of the host. Gene trees for each treeWAS-identified gene are shown in Fig. S4, which generally split loci into clades by most common host (*Vitis*, *Prunus* or *Coffea*).

### Detection of selection in the introduced population

The McDonald–Kreitman test was run on all core-genome genes, and in comparing the ancestral strains to the introduced strains, no genes showed an elevation of fixed non-synonymous substitutions between the two groups. Whilst many genes were found to have increases in non-synonymous substitutions, they were not fixed between groups, showing a lack of differentiation between the introduced strains and the CR strains.

Thirty-eight annotated genes and 33 hypothetical proteins were found to have sites under positive selection, identified via HyPhy’s SLAC program. SLAC identifies positive selection at a per codon scale across all samples included by calculating the ratio of non-synonymous to synonymous substitutions (dNdS) (Tables 1 and S2), with a Bonferroni-corrected *P* value<0.05, but not with a pairwise comparison *P*<0.05. In terms of molecular functions, the largest KEGG orthology groupings were for metabolism [15] and genetic information processing [10,25].

## Discussion

Information on *X. fastidiosa* subsp. *fastidiosa* infections in coffee in Central America remains limited. Although the disease is associated with few visible symptoms, persistent infections are observed in the field [26]. Our results are consistent with the possibility that a host jump to *Vitis* spp. may have been accompanied by changes reducing the capacity of US strains to infect *C. arabica*. However, the movement of the pathogen throughout the xylem vessels of *C. arabica* plants demonstrates a higher infectivity than expected in a fully resistant plant. In contrast, there was a reduction of positive-testing *C. arabica* plants over the course of the year after infection. This demonstrates lower infection persistence in *C. arabica* than in *V. vinifera,* where infections in a greenhouse are sustained post-inoculation, similar to what was observed in Purcell and Saunders [27]. This could demonstrate a reduction in the ability to create chronic infections in *C. arabica* by the *Vitis*-adapted strains. It is possible that the chronic infections of *X. fastidiosa* in *C. arabica* plants observed *in situ* may be caused by high rates of re-inoculation by insect vectors rather than strain-level variation in infectivity. That is not the case for subsp. *fastidiosa* in *V. vinifera*. Just one inoculation event is sufficient for high virulence and infectivity, and only cold winter temperatures have been known to cure infections that are otherwise chronic [28,29].

The virulence of the CA strains to *C. arabica* is not as high as to *V. vinifera*, as shown by the less severe symptoms in *C. arabica* in this study. Given that, this study does not offer evidence that the CA subsp. *fastidiosa* strains are generally more virulent than the ancestral strains in Central America. Whilst not a likely scenario, there was a possibility that instead of experiencing adaptation to a specific host, the introduced strains became generally more virulent, which has been hypothesized about the globally spreading subsp. *pauca* strain infecting olive in Italy [22]. We present hypervirulence as a possible scenario, but it is not supported by the data, and therefore, we were able to rule it out. Symptom development in infected *C. arabica* plants consisted of minor stunted growth, compared to the severe leaf scorch, matchstick petioles, shrivelled fruit and often plant death that occurs in *V. vinifera*.

Whilst not showing hypervirulence, the stunting of infected plants was significant in contrast to plants without bacterial infection. The interaction between infection status and date was also highly significant. Over the course of the study, as the infection waned, so did the stunting. These results may reflect an ongoing divergence process between populations adapted to different hosts. This supports the possibility that this system is in a transitional phase. Whilst strains in CR seem to be able to sometimes infect *Vitis* spp. but persistently infect *C. arabica*, CA strains are able to persistently infect *Vitis* spp. and only non-persistently infect * C. arabica*. These data are similar to those found on the genetic side – there is some evidence of population differentiation, but not fixed divisions between the two groups (speciation). There is also uncertainty around the potential of CR strains to infect *Vitis* spp. Although the isolates that have been tested so far were unable to sustain infection, there is much more genetic diversity of subsp. *fastidiosa* in CR than in the USA, and some isolates may indeed be infectious towards *Vitis* spp. In a previous study conducted in Brazil by Prado *et al*. [30], a *C. arabica* isolate caused disease in *C. arabica*, and it did not infect *Citrus sinensis*. Genetically, a * C. sinensis*-infecting clade emerged from a clade infecting *C. arabica* and subsequently lost the ability to infect *C. arabica*, indicating a host jump [31]. This may represent a parallel to the situation that we are observing here, with the introduction into the USA. Working with quarantine pathogens, however, makes these hypotheses difficult to interrogate due to legal requirements for facilities that are typically unavailable to researchers.

**Fig. 4.**
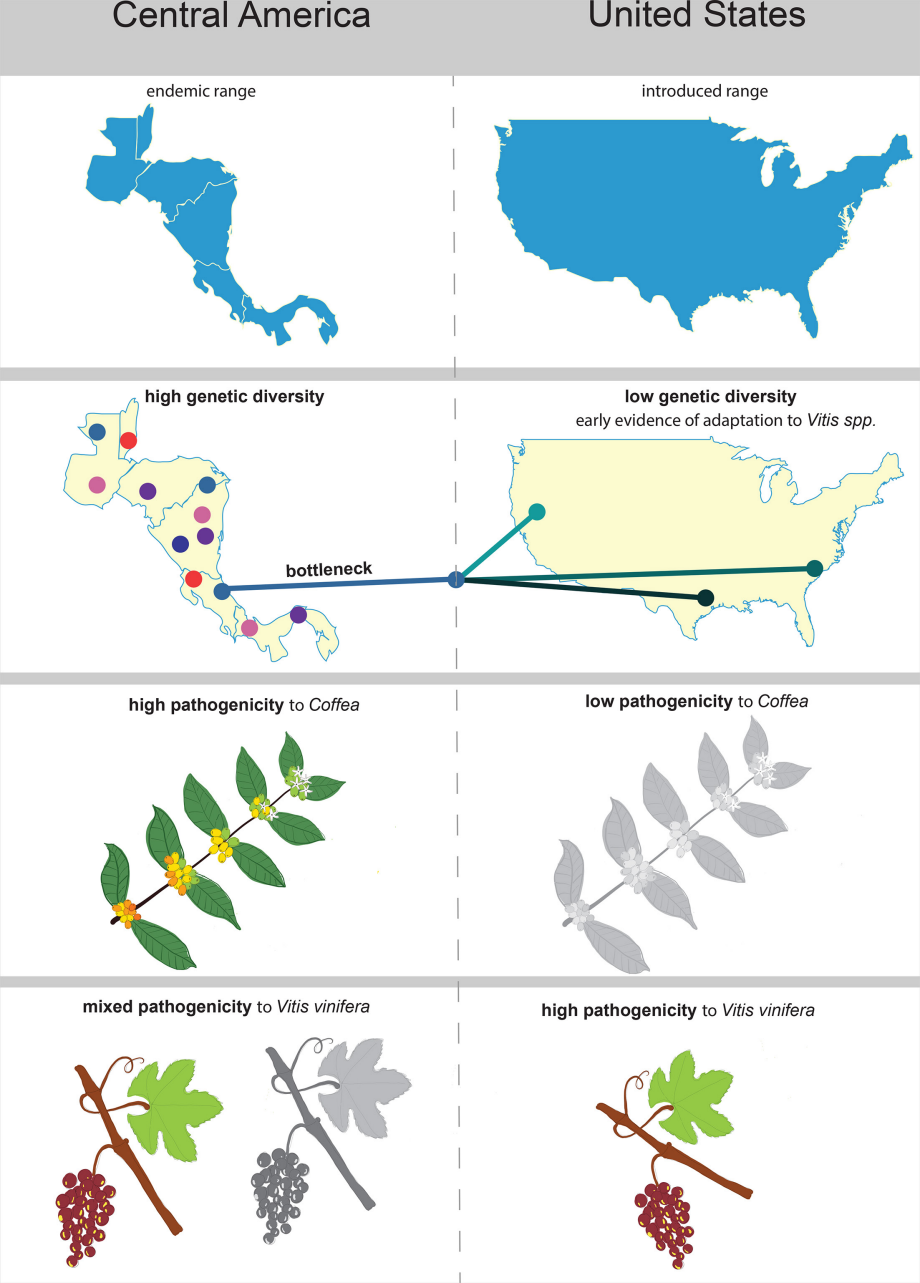
Conceptual model of the contrasting lifestyle of the pathogen *X. fastidiosa* subsp. *fastidiosa* in Central America in contrast to the USA. Central America is the endemic range of this subspecies, which has been shown through phylogenetics and relative diversity metrics. There is much higher genetic diversity in Central America, whereas in the USA, strains have radiated post-genetic bottleneck several hundred years ago. There is still very limited genetic diversity, even though many more strains have been sampled from the USA than from Central America. Strains from Central America have been documented to cause persistent infections in *C. arabica*, whereas the strains that were tested in CA were not able to persistently infect the same host, showing a reduction in pathogenicity. In an important host in the USA – *Vitis* spp., there are mixed reports of pathogenicity in Central America, with some studies showing no infection persistence, whilst others have reported field symptoms of Pierce’s disease. In the USA, all strains of subspecies *fastidiosa* that have been tested so far have high pathogenicity to the documented susceptible *Vitis* spp. (*Vitis labrusca* and *V. vinifera*).

The uniformly negative results of the McDonald–Kreitman test show that these populations have not fully diverged from each other. Other methods used detected probable genetic signatures of adaptation and found allele frequencies varying widely between the two groups. We were able to identify genes and SNPs associated with the host *Vitis* spp., as well as genes under positive selection in strains isolated from *Vitis* spp. These included both synonymous and non-synonymous variants. Although synonymous SNPs were initially thought to be neutral, growing evidence suggests that they can influence pathogen adaptation [32,33]. These genes included many hypothetical proteins; however, genes with known functions pertaining to infections by *X. fastidiosa* were also identified. These results collectively suggest that whilst the populations are beginning to differentiate, they are not yet genetically distinct. These changes in allele frequencies could be driven by selection, even if the changes have not reached complete fixation. This likely explains some of the ambiguity surrounding this introduction event in terms of host range. This genome-wide variation may reflect early signs of divergence associated with differences in host use (hypothesis summarized in Fig. 4).

The genes identified included those whose functions in *X. fastidiosa* have been previously investigated, whilst many still have unknown functions [34]. *ClpX*, the gene for the ATP-dependent protease ATP-binding subunit, was previously identified as being upregulated fourfold during the induction of biofilm formation [35]. *TolB* encodes for a translocation protein involved in membrane integrity and has also been shown to be important for biofilm development [36]. Mutations in the copper-related gene *copA* have been found to drastically alter copper tolerance in *X. fastidiosa*, which is vital for agricultural survival given the frequency of copper in treating fungal infections in the vineyard, a fungicide that has been in use since the eighteenth century [37,39]. It is possible that after a host jump into grapevines, it would be necessary for pathogens to survive higher levels of copper exposure. *DegP* has been found to be upregulated upon heat shock in *X. fastidiosa* [40]. These are amongst a suite of other genes that are both hypothetical proteins or just understudied in *X. fastidiosa* but show evidence of being involved in the process of this host and climate shift.

These genes are candidates for targeting in future experiments to determine their potential effects on host range and climate adaptation. Type IV pilus-related genes were detected by multiple methods as being under selection. Recent work has shown that these genes have duplicated and neofunctionalized many times over recent evolutionary history in *X. fastidiosa* and are also important to bacterial movement and natural competence [41]. Changes in these genes have been shown to differentially modulate virulence towards plant hosts by altering biofilm formation and affecting planktonic growth. Six genes within this suite – *pilT1*, *pilT2*, *pilA1*, *pilA2* and *pilY1* – were identified by either treeWAS or HyPhy based on elevated dN/dS ratios and the presence of SNPs associated with *Vitis* hosts. Notably, each programme highlighted different genes, suggesting methodological differences in detecting selection or, alternatively, multiple loci in the suite responding to selective pressures. Overall, many biofilm formation-related genes were identified. Biofilm formation is a physiological state essential for both virulence and colonization of insect vectors [42]. *pilA1*, *pilA2 and pilT* were knocked out in a recent paper, and findings showed varied but significant effects on motility, natural transformation rates and disease severity in tobacco plants (*Nicotiana tabacum*) [41]. In *X. fastidiosa*, gene regulation governs the transition between biofilm formation and motility, two states with distinct roles in the pathogen’s life cycle. Biofilms are critical for adhering to vector mouthparts and ensuring successful transmission, but they limit movement within the plant host, where the bacteria replicate more efficiently [42,43]. Thus, the regulation of biofilm production represents a potential mechanism by which *X. fastidiosa* could adapt to selective pressures from novel hosts through modulation of its virulence traits. Overall, these findings indicate that the populations have undergone some degree of genetic differentiation, though not to the extent of fixed divergence.

This study also includes one hypothesis that lies outside the general narrative of the introduction event and disease emergence, namely, the evaluation of infectivity of subsp. *multiplex* towards *C. arabica*, which to our knowledge has not been tested before. Whilst in CA, subsp. *multiplex* has never been found infecting grapevine in the field. It has been shown to generate non-persistent infections, similar to what we observed in *C. arabica*, in the greenhouse [8,44]. Recently, infections of grapevine by subsp. *multiplex* were detected in the field in VA, USA [45]. Whilst the subsp. *multiplex* infections were not highly virulent or as persistent as the subsp. *fastidiosa* strains, they were still able to infect the *C. arabica* plants transitorily. All three main subspecies of *X. fastidiosa* are able to infect *C. arabica* to some degree (see Fig. 1).

In conclusion, we have identified a suite of genes that are related to an *X. fastidiosa* host switch to *Vitis* spp. with a corresponding reduction in the ability of the pathogen to infect an ancestral host. Our findings support the hypothesis that the introduction event may have involved a shift in host use, potentially accompanied by reduced infectivity towards the ancestral host and improved compatibility with the novel host.

## Experimental procedures

### Coffee plant infections

**Fig. 5.**
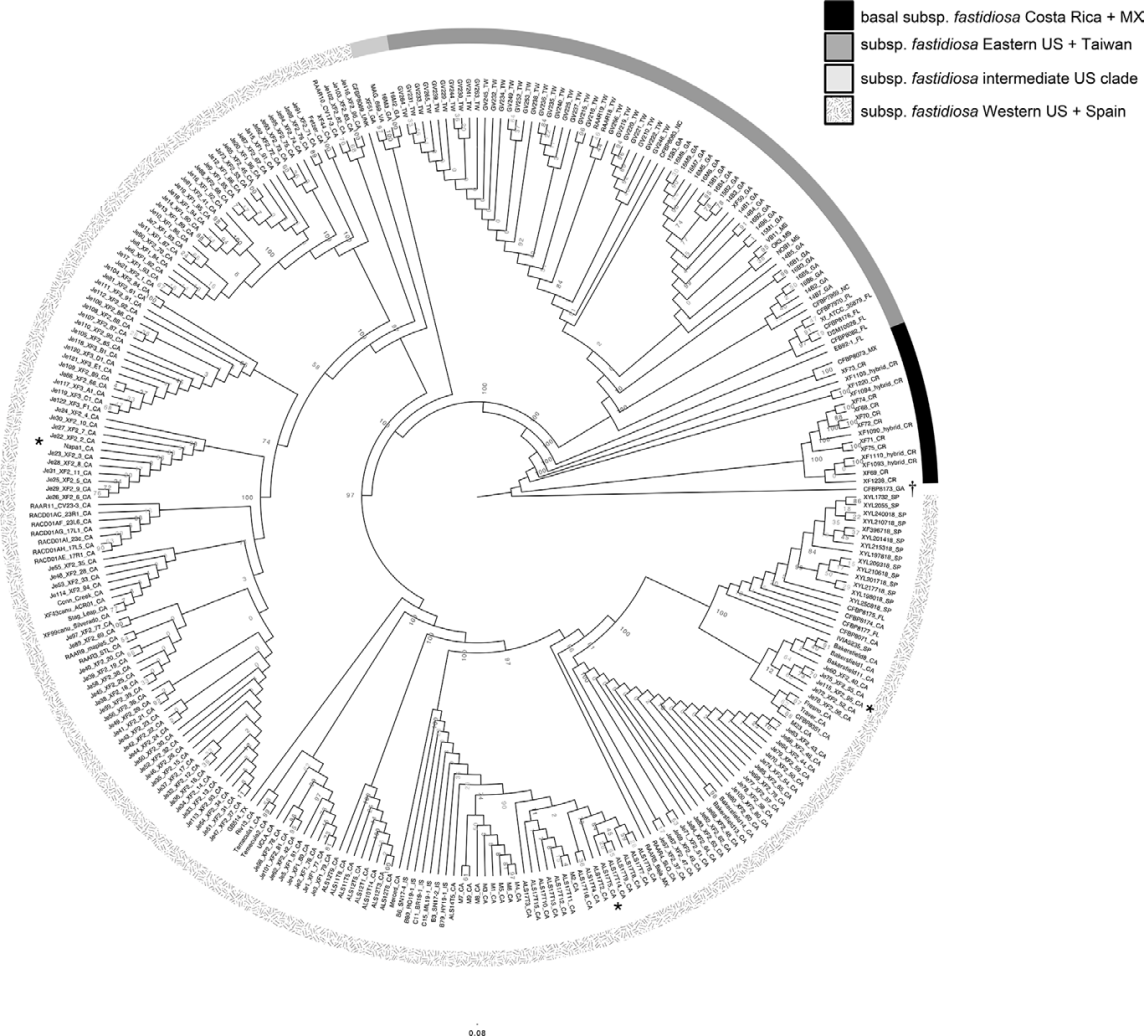
Cladogram of *X. fastidiosa* subsp. *fastidiosa* with one *X. fastidiosa* subsp. *multiplex* sample, CFBP8173_GA as the outgroup, denoted with a dagger (†). At the base of the tree are samples from CR, as well as one isolate from Mexico (MX) (black). The introduced clade includes a clade from the Eastern USA and an introduction into Taiwan (dark grey), an intermediate clade between the US east and west coasts (light grey) and the clade on the west coast which includes introductions into Spain and Israel (speckles). Bootstrap support is shown numerically at each node, and the substitution rate is shown with a bar below the tree. The three subsp. *fastidiosa* strains used for the inoculation experiment are denoted with an asterisk (*).

In March 2022, 120 coffee plants (*C. arabica*) were divided into equal groups and inoculated with either a buffer control or one of four CA strains of *X. fastidiosa*, subsp. *fastidiosa*: Napa1 (from *V. vinifera*, Napa co., CA, isolated 2015), ALS17T5 (from * P. dulcis*, San Joaquin co., CA, isolated 2020), Je115 (from *V. vinifera*, Bakersfield, CA, isolated 2016) subsp. *multiplex* ALS15T2 (from *P. dulcis*, Claribel, CA, isolated 2020), or the sterile SCP buffer control [46]. Whilst Napa1 and Je115 were both isolated from *V. vinifera*, they are from different climates and clades of *X. fastidiosa* and might have experienced distinctive selective regimes such as mean annual temperatures (Napa1 was isolated in Napa county, mean annual temp=14.3 °C, and Je115 was isolated in Bakersfield county, mean annual temp=16.4 °C) [8,47]. ALS17T5 was isolated from a symptomatic almond plant and is in one of several clades of strains infecting only almond trees. However, this clade is nested within other clades that infect *Vitis* spp. (see Fig. 5).

Disease-free *C. arabica* plants (60 cv. Geisha, 60 cv. Red Catuaí; 12 replicates per treatment) were donated from Frinj coffee company for this experiment. As positive controls, 20 *V*. *vinifera* cv. Chardonnay (four per strain) were inoculated, as well as 50 5-week-old *H. annuus* grown from seed. *V. vinifera* is known to be highly susceptible to subspecies *fastidiosa* but not susceptible to subspecies *multiplex*, whilst *H. annuus* is susceptible to both subspecies [48,49]. Cell suspensions were prepared by suspending 1-week-old cells grown on a solid medium in SCP buffer just prior to inoculation. Each suspension was made by scraping ten streaks of 20 µl into 1 ml of buffer. Inoculations were conducted using two 10 µl drops of inoculum and a 00-size entomological pin used to pinprick through the bead of cell suspension several times until the inoculum was absorbed into the plant xylem. Inoculations were conducted on small plants with typically three full-leaf pairs, and two inoculum beads were placed just above and below the centre leaf pair. Plants were not watered on the morning of inoculation to optimize absorption of the inoculum into the xylem vessels.

Symptom measurements took place in August, September, October and December 2022 and January and April 2023, following the March 2022 inoculation. In August and September 2022, all internode lengths along the main stem of the plant were measured along with the heights of each plant. In October, December, January and April, only the total plant heights were measured to detect stunting. Plants were also visually assessed for foliar scorching.

Control and experimental plants were tested for the presence of *X. fastidiosa* via qPCR or culturing. DNA was extracted using a DNeasy plant mini kit (Qiagen) and then quantified using qPCR with a primer pair targeting the gene encoding *recF*, RecF1_F+R; for qPCR protocol, see Sicard *et al*. [50]. qPCR was run in duplicate with positive and negative controls on each plate; all samples with quantification cycle values of 37 or higher were considered ‘undetected’ and were considered negative. Attempts to isolate bacterial strains were conducted using ~0.1 g of petiole tissue, or the entire petiole and midrib of a *C. arabica* leaf. Samples were surface sterilized, chopped, ground in a Polytron and plated on a PWG medium using the methods described in Hill and Purcell [51].

On 3 April 2022, petioles that were directly above the inoculation site were sampled from all 50 *H*. *annuus* plants, and *X. fastidiosa* populations were measured via both qPCR and cell culturing. On July 14, one leaf from the second leaf pair above the inoculation point was used for detection via qPCR. On August 1, 15 and 18, one leaf from the second leaf pair above the inoculation point was cultured from each *C. arabica* plant. On 4 October 2022, the opposite leaf was taken for qPCR. Whilst *Vitis* spp. are not as susceptible to infections from subsp. *multiplex*, typically, there is some detectable infection; however, those infections have 10- to 100-fold lower population sizes than infections by subsp. *fastidiosa* [44].

In February to April 2023, we isolated samples from the leaves using a different method due to the loss of leaves from the ~8 cm above the inoculation point. The lowest eight leaves were instead collected, and the petioles were pooled for sterilization, tissue grinding and plating.

### Statistical analysis of inoculation data

All analyses were performed using R statistical software v4.2.3 [52]. Linear mixed models were built using the package lme4 v1.1-32 to add a random effect to account for repeated sampling of the plants over the course of the experiment [53]. (a) As analyses of virulence, we tested the fixed effects of the interaction between infection status (a binary result of testing positive at any of the four time points) and sampling date, as well as those variables on their own, and inoculation treatment, on both internode length, and (b, c) separately on plant height (split into two models by variety), with a random effect of plant identity using the lmer function of lme4. (d) As an analysis of infection persistence via detection assays, we tested the effects of variety and the interaction between treatment and sampling date on the count of plants that tested positive using a generalized linear mixed model with binomial error using the package glmmTMB v.1.1.7 and the function glmmTMB [54].

### Whole-genome dataset

Sequences used for this analysis were assembled in-house from prior publications or downloaded from NCBI and annotated using Prokka v1.14.5 [55]. All publicly available whole-genome sequences from *X. fastidiosa* subsp. *fastidiosa* were used, as well as one sequence from subsp. *multiplex* which was used as an outgroup for phylogenetic analyses. A list of the metadata for all strains and sequence information can be found in Table S1.

### Alignment and phylogenetics

Alignment and phylogenetics were conducted using the pipeline from Donegan *et al*. [56] (https://github.com/MonicaDonegan/aDNA-xylella-herbarium). The annotations for each sequence were run using the alignment program Panaroo v1.2.10 [57], which was run in strict clean mode and used to create the pan and core genomes, as well as the core gene alignments. Core genome alignments (with and without the subsp. *multiplex* outgroup CFBP8173) were run through ClonalFrameML v1.12 [58] to identify recombinant regions, which were removed using an in-house Python script. An SNP alignment was extracted from the non-recombinant alignment using snp-sites v2.5.1 [59]. The non-recombinant SNP alignment was then used to generate a phylogenetic tree in RAxML v8.2.11 [60] using the GTR cat parameters and 100 iterations.

### GWASs

treeWAS v1.1 [61] was run on core genome SNPs to quantify SNPs that were significantly associated with strains isolated from the genus *Vitis*. treeWAS was designed for microbial GWASs specifically to deal with clonal populations that frequently recombine. Scoary v1.6.16 [62] was also run on both the core genome SNPs (without recombination removed) as well as the gene presence-absence file (generated using Panaroo and snp-sites) to correlate genome content amongst strains isolated from plants in the genus *Vitis*. Most strains were isolated from *V. vinifera* aside from two isolates from *Vitis rotundifolia* and three unidentified *Vitis* spp. Scoary uses a phylogeny in order to remove lineage-specific interdependencies and offers both a multiple hypothesis-corrected *P* value as well as a phylogenetically corrected *P* value. treeWAS was also run to compare strains from the clade including the US strains in contrast to all outgroup strains.

### Detection of regions under positive selection

To detect whether there was evidence of either positive or negative selection via dN/dS ratio calculations, the SLAC test from HyPhy v2.5.40 [63] was used on only *Vitis* spp. samples. SLAC is a site-specific tool that calculates substitution rates at individual codons given the alignment and phylogeny. Core gene alignments were built using Panaroo and subsequently aligned based on protein-coding sequences and trimmed to prepare for input using Macse v10.02 [64]. Individual core gene trees were constructed using RAxML. These inputs were collectively used to run SLAC. The same processing was then used on run-on gene alignments of the entire dataset (*Vitis* and non-*Vitis*). After strains with >50% gaps were removed (gene-by-gene), the McDonald–Kreitman test [65] was also run to test for fixed non-synonymous substitutions that differentiate the ancestral population from the introduced strains. These tests were run using the R package PopGenome v2.7.5 [66].

## Supplementary material

10.1099/mgen.0.001447 Uncited Fig. S1.

## Abbreviations

CA: California
CR: Costa Rica
GWAS: genome-wide association study
MX: Mexico
PD: Pierce's Disease
SCP: succinate-citrate-phosphate
SLAC: Single-Likelihood Ancestor Counting
