# Supplementary material for: A pathogen of good taste: genetics of a bacterial host jump of the plant pathogen Xylella fastidiosa from coffee to wine grapes
Source: Microb Genom. 2025 Jul 17;11(7):001447. doi: 10.1099/mgen.0.001447 (PMC12284412; doi:10.1099/mgen.0.001447)
Supplement: Uncited Fig. S1. [file mgen-11-01447-s001.pdf]

Supplemental Legends

Table S1: Metadata with sequence information. The metadata sheet includes information on all sequences used in this study. It includes the sequence name, host information, isolation year, geographic origin, and biosample information.

Strain Name	Host	Isolation year	Geographic Origin	Accession
CFBP8071	*P. dulcis*	1987	USA, California	SAMN07998881
M23	*P. dulcis*	2003	USA, California	SAMN02598408
B3 (SN17-2)	*P. dulcis*	2017	Israel	SAMN26809501
B6 (SN17-4)	*P. dulcis*	2017	Israel	SAMN26809502
XYL197818	*P. dulcis*	2018	Spain	SAMN14008175
XYL198018	*P. dulcis*	2018	Spain	SAMN14052381
XYL201418	*P. dulcis*	2018	Spain	SAMN14052501
XYL201718	*P. dulcis*	2018	Spain	SAMN14052502
XYL209318	*P. dulcis*	2018	Spain	SAMN14052504
XYL210618	*P. dulcis*	2018	Spain	SAMN14052529
XYL210718	*P. dulcis*	2018	Spain	SAMN14052539
B79 (HY19-1)	*P. dulcis*	2019	Israel	SAMN26809503
B80 (RO19-1)	*P. dulcis*	2019	Israel	SAMN26809504
C11 (BR19-1)	*P. dulcis*	2019	Israel	SAMN26809505
C12 (BL19-1)	*P. dulcis*	2019	Israel	PRJNA817832
C15 (ML19-1)	*P. dulcis*	2019	Israel	SAMN26809506
M1 (Madera-1)	*P. dulcis*	2019	USA, California	SAMN41527522
M2 (Madera-2)	*P. dulcis*	2019	USA, California	SAMN34030392
M3 (Madera-3)	*P. dulcis*	2019	USA, California	SAMN34030393
M4 (Madera-4)	*P. dulcis*	2019	USA, California	SAMN34030394
M5 (Madera-5)	*P. dulcis*	2019	USA, California	SAMN34030395
M6 (Madera-6)	*P. dulcis*	2019	USA, California	SAMN34030396
M7 (Madera-7)	*P. dulcis*	2019	USA, California	SAMN34030397
M8 (Madera-8)	*P. dulcis*	2019	USA, California	SAMN34030398
M9 (Madera-9)	*P. dulcis*	2019	USA, California	SAMN34030399
ALS10T14	*P. dulcis*	2020	USA, California	SAMN23611068
ALS11T5	*P. dulcis*	2020	USA, California	SAMN23611072
ALS11T8	*P. dulcis*	2020	USA, California	SAMN23611073
ALS12T1	*P. dulcis*	2020	USA, California	SAMN23611074
ALS12T3	*P. dulcis*	2020	USA, California	SAMN23611079
ALS12T5	*P. dulcis*	2020	USA, California	SAMN23611081
ALS12T8	*P. dulcis*	2020	USA, California	SAMN23611082
ALS12T9	*P. dulcis*	2020	USA, California	SAMN23611083
ALS14T5	*P. dulcis*	2020	USA, California	SAMN23611086
ALS17T10	*P. dulcis*	2020	USA, California	SAMN23611093
ALS17T11	*P. dulcis*	2020	USA, California	SAMN23611094
ALS17T12	*P. dulcis*	2020	USA, California	SAMN23611095
ALS17T13	*P. dulcis*	2020	USA, California	SAMN23611096
ALS17T14	*P. dulcis*	2020	USA, California	SAMN23611097
ALS17T15	*P. dulcis*	2020	USA, California	SAMN23611098
ALS17T16	*P. dulcis*	2020	USA, California	SAMN23611099
ALS17T2	*P. dulcis*	2020	USA, California	SAMN23611100
ALS17T3	*P. dulcis*	2020	USA, California	SAMN23611101
ALS17T4	*P. dulcis*	2020	USA, California	SAMN23611102
ALS17T5	*P. dulcis*	2020	USA, California	SAMN23611103
ALS17T6	*P. dulcis*	2020	USA, California	SAMN23611104
ALS17T7	*P. dulcis*	2020	USA, California	SAMN23611105
ALS17T8	*P. dulcis*	2020	USA, California	SAMN23611106
ALS17T9	*P. dulcis*	2020	USA, California	SAMN23611107
Fresno	*P. dulcis*	1995	USA, California	SAMN27988137
CFBP8073	*C. canephora*	2012	Mexico	SAMN04075659
XF69	*C. arabica*	2016	Costa Rica	SAMN40622577
XF70	*C. arabica*	2016	Costa Rica	SAMN12994818
XF71	*C. arabica*	2016	Costa Rica	SAMN12994819
XF72	*C. arabica*	2016	Costa Rica	SAMN12994820
XF73	*C. arabica*	2016	Costa Rica	SAMN12994821
XF74	*C. arabica*	2016	Costa Rica	SAMN12994822

XF75	*C. arabica*	2016	Costa Rica	SAMN12994823
XF1090	*C. arabica*	2017	Costa Rica	SAMN12994824
XF1093	*C. arabica*	2017	Costa Rica	SAMN12994825
XF1105	*C. arabica*	2017	Costa Rica	SAMN12994827
XF1220	*C. arabica*	2018	Costa Rica	SAMN40622575
XF1238	*C. arabica*	2018	Costa Rica	SAMN40622576
EB92-1	*Sambucus sp.*	1992	USA, Florida	SAMN02471770
NOB1	*V. rotundifolia*	2019	USA, Mississippi	SAMN14675132
CFBP7969	*V. rotundifolia*	1985	USA, North Carolina	SAMN07998892
CFBP7970	*V. sp.*	1987	USA, Florida	SAMN07998897
TPD3	*V. sp.*	2012	Taiwan	SAMN12097273
TPD4	*V. sp.*	2012	Taiwan	SAMN12097274
CFBP8083	*V. vinifera*	1985	USA, North Carolina	SAMN27988126
DSM10026	*V. vinifera*	1987	USA, Florida	SAMN05660380
Xf_ATCC_35879	*V. vinifera*	1987	USA, Florida	SAMN02997312
CFBP8351	*V. vinifera*	1993	USA, California	SAMN07999362
SLO	*V. vinifera*	1994	USA, California	SAMN09941414
STL	*V. vinifera*	1998	USA, California	SAMN09941416
Temecula1	*V. vinifera*	1998	USA, California	SAMN02603844
RAAR5_Baja	*V. vinifera*	2001	Mexico	SAMN09941403
Stags_Leap	*V. vinifera*	2005	USA, California	SAMN24371048
GB514	*V. vinifera*	2006	USA, Texas	SAMN02603754
Xf50 (WM1-1)	*V. vinifera*	2009	USA, Georgia	SAMN09941434
Xf51 (CCPM1)	*V. vinifera*	2010	USA, Georgia	SAMN09941435
RAAR10_CV17-3	*V. vinifera*	2011	USA, California	SAMN26366572
RAAR11_CV23	*V. vinifera*	2011	USA, California	SAMN09941404
Xf43_ACR-01	*V. vinifera*	2011	USA, California	SAMN09941432
RACD01AC (23R1)	*V. vinifera*	2012	USA, California	SAMN09941408
RACD01AE (17R1)	*V. vinifera*	2012	USA, California	SAMN09941409
RACD01AF (23L6)	*V. vinifera*	2012	USA, California	SAMN09941410
RACD01AG (17L1)	*V. vinifera*	2012	USA, California	SAMN09941411
RACD01AH (17L5)	*V. vinifera*	2012	USA, California	SAMN09941412
RACD01AI (23c)	*V. vinifera*	2012	USA, California	SAMN09941413
14B1	*V. vinifera*	2014	USA, Georgia	SAMN15732826
14B2	*V. vinifera*	2014	USA, Georgia	SAMN15732827
14B3	*V. vinifera*	2014	USA, Georgia	SAMN15732828
14B4	*V. vinifera*	2014	USA, Georgia	SAMN15732829
14B5	*V. vinifera*	2014	USA, Georgia	SAMN15732830
14B6	*V. vinifera*	2014	USA, Georgia	SAMN15732831
14B7	*V. vinifera*	2014	USA, Georgia	SAMN15732832
GV210	*V. vinifera*	2015	Taiwan	SAMN18344333
Napa1	*V. vinifera*	2015	USA, California	SAMN09941406
15B1	*V. vinifera*	2015	USA, Georgia	SAMN15732833
15B2	*V. vinifera*	2015	USA, Georgia	SAMN15732834
15B3	*V. vinifera*	2015	USA, Georgia	SAMN15732835
15M1	*V. vinifera*	2015	USA, Georgia	SAMN15732837
Bakersfield-1	*V. vinifera*	2016	USA, California	SAMN11914553
Bakersfield-8	*V. vinifera*	2016	USA, California	SAMN16582176
Je1	*V. vinifera*	2016	USA, California	SAMN10358662
Je10	*V. vinifera*	2016	USA, California	SAMN10358663
Je100	*V. vinifera*	2016	USA, California	SAMN10358664
Je101	*V. vinifera*	2016	USA, California	SAMN10358665
Je102	*V. vinifera*	2016	USA, California	SAMN10358666
Je103	*V. vinifera*	2016	USA, California	SAMN10358667
Je104	*V. vinifera*	2016	USA, California	SAMN10358668
Je105	*V. vinifera*	2016	USA, California	SAMN10358669
Je106	*V. vinifera*	2016	USA, California	SAMN10358670
Je107	*V. vinifera*	2016	USA, California	SAMN10358671
Je108	*V. vinifera*	2016	USA, California	SAMN10358672
Je109	*V. vinifera*	2016	USA, California	SAMN10358673
Je11	*V. vinifera*	2016	USA, California	SAMN10358674
Je110	*V. vinifera*	2016	USA, California	SAMN10358675
Je111	*V. vinifera*	2016	USA, California	SAMN10358676
Je112	*V. vinifera*	2016	USA, California	SAMN10358677

Je113	*V. vinifera*	2016	USA, California	SAMN10358678
Je114	*V. vinifera*	2016	USA, California	SAMN10358679
Je115	*V. vinifera*	2016	USA, California	SAMN10358680
Je116	*V. vinifera*	2016	USA, California	SAMN10358681
Je117	*V. vinifera*	2016	USA, California	SAMN10358682
Je118	*V. vinifera*	2016	USA, California	SAMN10358683
Je119	*V. vinifera*	2016	USA, California	SAMN10358684
Je12	*V. vinifera*	2016	USA, California	SAMN10358685
Je120	*V. vinifera*	2016	USA, California	SAMN10358686
Je121	*V. vinifera*	2016	USA, California	SAMN10358687
Je122	*V. vinifera*	2016	USA, California	SAMN10358688
Je13	*V. vinifera*	2016	USA, California	SAMN10358689
Je14	*V. vinifera*	2016	USA, California	SAMN10358690
Je15	*V. vinifera*	2016	USA, California	SAMN10358691
Je16	*V. vinifera*	2016	USA, California	SAMN10358692
Je17	*V. vinifera*	2016	USA, California	SAMN10358693
Je18	*V. vinifera*	2016	USA, California	SAMN10358694
Je19	*V. vinifera*	2016	USA, California	SAMN10358695
Je2	*V. vinifera*	2016	USA, California	SAMN10358696
Je20	*V. vinifera*	2016	USA, California	SAMN10358697
Je21	*V. vinifera*	2016	USA, California	SAMN10358698
Je22	*V. vinifera*	2016	USA, California	SAMN10358699
Je23	*V. vinifera*	2016	USA, California	SAMN10358700
Je24	*V. vinifera*	2016	USA, California	SAMN10358701
Je25	*V. vinifera*	2016	USA, California	SAMN10358702
Je26	*V. vinifera*	2016	USA, California	SAMN10358703
Je27	*V. vinifera*	2016	USA, California	SAMN10358704
Je28	*V. vinifera*	2016	USA, California	SAMN10358705
Je29	*V. vinifera*	2016	USA, California	SAMN10358706
Je3	*V. vinifera*	2016	USA, California	SAMN10358707
Je30	*V. vinifera*	2016	USA, California	SAMN10358708
Je31	*V. vinifera*	2016	USA, California	SAMN10358709
Je32	*V. vinifera*	2016	USA, California	SAMN10358710
Je33	*V. vinifera*	2016	USA, California	SAMN10358711
Je34	*V. vinifera*	2016	USA, California	SAMN10358712
Je35	*V. vinifera*	2016	USA, California	SAMN10358713
Je36	*V. vinifera*	2016	USA, California	SAMN10358714
Je37	*V. vinifera*	2016	USA, California	SAMN10358715
Je38	*V. vinifera*	2016	USA, California	SAMN10358716
Je39	*V. vinifera*	2016	USA, California	SAMN10358717
Je4	*V. vinifera*	2016	USA, California	SAMN10358718
Je40	*V. vinifera*	2016	USA, California	SAMN10358719
Je41	*V. vinifera*	2016	USA, California	SAMN10358720
Je42	*V. vinifera*	2016	USA, California	SAMN10358721
Je43	*V. vinifera*	2016	USA, California	SAMN10358722
Je44	*V. vinifera*	2016	USA, California	SAMN10358723
Je45	*V. vinifera*	2016	USA, California	SAMN10358724
Je46	*V. vinifera*	2016	USA, California	SAMN10358725
Je47	*V. vinifera*	2016	USA, California	SAMN10358726
Je48	*V. vinifera*	2016	USA, California	SAMN10358727
Je49	*V. vinifera*	2016	USA, California	SAMN10358728
Je5	*V. vinifera*	2016	USA, California	SAMN10358729
Je50	*V. vinifera*	2016	USA, California	SAMN10358730
Je51	*V. vinifera*	2016	USA, California	SAMN10358731
Je52	*V. vinifera*	2016	USA, California	SAMN10358732
Je53	*V. vinifera*	2016	USA, California	SAMN10358733
Je54	*V. vinifera*	2016	USA, California	SAMN10358734
Je55	*V. vinifera*	2016	USA, California	SAMN10358735
Je56	*V. vinifera*	2016	USA, California	SAMN10358736
Je57	*V. vinifera*	2016	USA, California	SAMN10358737
Je58	*V. vinifera*	2016	USA, California	SAMN10358738
Je59	*V. vinifera*	2016	USA, California	SAMN10358739
Je6	*V. vinifera*	2016	USA, California	SAMN10358740
Je60	*V. vinifera*	2016	USA, California	SAMN10358741
Je61	*V. vinifera*	2016	USA, California	SAMN10358742
Je62	*V. vinifera*	2016	USA, California	SAMN10358743

Je63	*V. vinifera*	2016	USA, California	SAMN10358744
Je64	*V. vinifera*	2016	USA, California	SAMN10358745
Je65	*V. vinifera*	2016	USA, California	SAMN10358746
Je66	*V. vinifera*	2016	USA, California	SAMN10358747
Je67	*V. vinifera*	2016	USA, California	SAMN10358748
Je68	*V. vinifera*	2016	USA, California	SAMN10358749
Je69	*V. vinifera*	2016	USA, California	SAMN10358750
Je7	*V. vinifera*	2016	USA, California	SAMN10358751
Je70	*V. vinifera*	2016	USA, California	SAMN10358752
Je71	*V. vinifera*	2016	USA, California	SAMN10358753
Je72	*V. vinifera*	2016	USA, California	SAMN10358754
Je73	*V. vinifera*	2016	USA, California	SAMN10358755
Je74	*V. vinifera*	2016	USA, California	SAMN10358756
Je75	*V. vinifera*	2016	USA, California	SAMN10358757
Je76	*V. vinifera*	2016	USA, California	SAMN10358758
Je77	*V. vinifera*	2016	USA, California	SAMN10358759
Je78	*V. vinifera*	2016	USA, California	SAMN10358760
Je79	*V. vinifera*	2016	USA, California	SAMN10358761
Je8	*V. vinifera*	2016	USA, California	SAMN10358762
Je80	*V. vinifera*	2016	USA, California	SAMN10358763
Je81	*V. vinifera*	2016	USA, California	SAMN10358764
Je82	*V. vinifera*	2016	USA, California	SAMN10358765
Je83	*V. vinifera*	2016	USA, California	SAMN10358766
Je84	*V. vinifera*	2016	USA, California	SAMN10358767
Je85	*V. vinifera*	2016	USA, California	SAMN10358768
Je86	*V. vinifera*	2016	USA, California	SAMN10358769
Je87	*V. vinifera*	2016	USA, California	SAMN10358770
Je88	*V. vinifera*	2016	USA, California	SAMN10358771
Je89	*V. vinifera*	2016	USA, California	SAMN10358772
Je9	*V. vinifera*	2016	USA, California	SAMN10358773
Je90	*V. vinifera*	2016	USA, California	SAMN10358774
Je91	*V. vinifera*	2016	USA, California	SAMN10358775
Je92	*V. vinifera*	2016	USA, California	SAMN10358776
Je93	*V. vinifera*	2016	USA, California	SAMN10358777
Je94	*V. vinifera*	2016	USA, California	SAMN10358778
Je95	*V. vinifera*	2016	USA, California	SAMN10358779
Je96	*V. vinifera*	2016	USA, California	SAMN10358780
Je97	*V. vinifera*	2016	USA, California	SAMN10358781
Je98	*V. vinifera*	2016	USA, California	SAMN10358782
Je99	*V. vinifera*	2016	USA, California	SAMN10358783
16B1	*V. vinifera*	2016	USA, Georgia	SAMN15732838
16B2	*V. vinifera*	2016	USA, Georgia	SAMN15732839
16B3	*V. vinifera*	2016	USA, Georgia	SAMN15732840
16B4	*V. vinifera*	2016	USA, Georgia	SAMN15732841
16B5	*V. vinifera*	2016	USA, Georgia	SAMN15732842
16B6	*V. vinifera*	2016	USA, Georgia	SAMN15732836
16M2	*V. vinifera*	2016	USA, Georgia	SAMN15732843
16M3	*V. vinifera*	2016	USA, Georgia	SAMN15732844
16M5	*V. vinifera*	2016	USA, Georgia	SAMN15732845
16M6	*V. vinifera*	2016	USA, Georgia	SAMN15732846
16M7	*V. vinifera*	2016	USA, Georgia	SAMN15732847
16M8	*V. vinifera*	2016	USA, Georgia	SAMN15732848
16M9	*V. vinifera*	2016	USA, Georgia	SAMN15732849
XYL1732	*V. vinifera*	2017	Spain	SAMN09767243
XYL2055	*V. vinifera*	2017	Spain	SAMN09767242
GV215	*V. vinifera*	2017	Taiwan	SAMN18344334
GV216	*V. vinifera*	2017	Taiwan	SAMN18344335
GV219	*V. vinifera*	2017	Taiwan	SAMN18344336
GV220	*V. vinifera*	2017	Taiwan	SAMN18344337
GV221	*V. vinifera*	2017	Taiwan	SAMN18344338
GV222	*V. vinifera*	2017	Taiwan	SAMN18344339
GV225	*V. vinifera*	2017	Taiwan	SAMN18344340
GV229	*V. vinifera*	2017	Taiwan	SAMN18344341
Bakersfield-11	*V. vinifera*	2017	USA, California	SAMN16582177
Bakersfield-13	*V. vinifera*	2017	USA, California	SAMN16582178
Bakersfield-14	*V. vinifera*	2017	USA, California	SAMN16582179

XF396718	*V. vinifera*	2018	Spain	SAMN14052556
XYL215318	*V. vinifera*	2018	Spain	SAMN14052541
XYL217718	*V. vinifera*	2018	Spain	SAMN14052551
XYL240018	*V. vinifera*	2018	Spain	SAMN14052552
XYL250818	*V. vinifera*	2018	Spain	SAMN14052553
GV230	*V. vinifera*	2018	Taiwan	SAMN15783671
GV231	*V. vinifera*	2018	Taiwan	SAMN18344342
GV232	*V. vinifera*	2018	Taiwan	SAMN18344343
GV233	*V. vinifera*	2018	Taiwan	SAMN18344344
GV234	*V. vinifera*	2018	Taiwan	SAMN18344345
GV235	*V. vinifera*	2018	Taiwan	SAMN18344346
GV236	*V. vinifera*	2018	Taiwan	SAMN18344347
GV237	*V. vinifera*	2018	Taiwan	SAMN18344348
GV238	*V. vinifera*	2018	Taiwan	SAMN18344349
GV239	*V. vinifera*	2018	Taiwan	SAMN18344350
GV240	*V. vinifera*	2018	Taiwan	SAMN18344351
GV241	*V. vinifera*	2018	Taiwan	SAMN18344352
GV244	*V. vinifera*	2018	Taiwan	SAMN18344353
GV245	*V. vinifera*	2018	Taiwan	SAMN18344354
GV248	*V. vinifera*	2019	Taiwan	SAMN18344355
GV249	*V. vinifera*	2019	Taiwan	SAMN18344356
GV252	*V. vinifera*	2019	Taiwan	SAMN18344357
GV253	*V. vinifera*	2019	Taiwan	SAMN18344358
GV263	*V. vinifera*	2019	Taiwan	SAMN18344359
GV264	*V. vinifera*	2019	Taiwan	SAMN18344360
GV265	*V. vinifera*	2019	Taiwan	SAMN18344361
GV266	*V. vinifera*	2019	Taiwan	SAMN18344362
OK3	*V. vinifera*	2019	USA, Mississippi	SAMN14675133
VB11	*V. vinifera*	2019	USA, Mississippi	SAMN14675134
MAG_669	*V. vinifera*	2020	USA, Virginia	SAMN22645440
Conn_Creek	*V. vinifera*	1995	USA, California	SAMN27988135
Fetzer	*V. vinifera*	1995	USA, California	SAMN27988136
Merced	*V. vinifera*	1995	USA, California	SAMN27988138
Temecula2	*V. vinifera*	1995	USA, California	SAMN27988139
Traver	*V. vinifera*	1995	USA, California	SAMN27988140
UCLA	*V. vinifera*	Pre 1982	USA, California	SAMN27988141
CFBP8069	*V. vinifera*	Pre 1989	USA, Florida	SAMN27988122
CFBP8174	*V. vinifera*	Pre 1981	USA, California	SAMN27988129
Xf44_Hopland	*V. vinifera*	1990-1999	USA, California	SAMN09941433
Xf99_Silverado	*V. vinifera*	1990-1999	USA, California	SAMN09941436
CFBP8175	*V. vinifera*	1985	USA, Florida	SAMN27988130
CFBP8176	*V. vinifera*	1985	USA, Florida	SAMN27988131
CFBP8177	*V. vinifera*	1985	USA, Florida	SAMN27988132
XF68	*Psidium sp.*	2012	Costa Rica	SAMN12994817
maple5	*Acer sp.*	2001	USA, California	SAMN09941405
XF1094	*Vinca sp.*	2017	Costa Rica	SAMN12994826
XF1110	*Vinca sp.*	2017	Costa Rica	SAMN12994828
CFBP8173	*Prunus domestica*	1983	USA, Georgia	SAMN27988128
CFBP8082	*Ambrosia artemisiifolia*	1983	USA, Florida	SAMN07999361
IVIA5235	*Prunus avium*	2017	Spain	SAMN09925804
Riv13	*Cercis occidentalis*	2006	USA, California	SAMN24371049

Table S2: HyPhy SLAC results on grape only samples, list of genes under positive selection. Only named genes (not the 33 significant hypothetical proteins) are included in this table. Annotations included are those found using Prokka v1.14.5.

Gene	Annotation	Kegg Orthology
acnM	Aconitate hydratase A	Metabolism
apaG	Protein ApaG	Unclassified: signaling and cellular processes
atpF	ATP synthase subunit b	Metabolism
clpX	ATP-dependent Clp protease ATP-binding subunit ClpX	genetic information processing
copA~ftsP	Copper resistance protein A;Cell division protein FtsP	Translocases
dapE	Succinyl-diaminopimelate desuccinylase	Metabolism
dcd	dCTP deaminase	Metabolism
ddlB_1~ddlB_2~ddl	D-alanine--D-alanine ligase B	Metabolism
degP	Periplasmic serine endoprotease DegP	Environmental Information Processing
dgkA	Diacylglycerol kinase	Metabolism
fmt	Methionyl-tRNA formyltransferase	Metabolism
gpsA	Glycerol-3-phosphate dehydrogenase [NAD(P)+]	Metabolism
gyrB	DNA gyrase subunit B	Protein families: genetic information processing
kdsD	Arabinose 5-phosphate isomerase KdsD	Metabolism
lacF	Lactose transport system permease protein LacF	Metabolism
leuB	3-isopropylmalate dehydrogenase	Metabolism
lpoA	Penicillin-binding protein activator LpoA	Poorly characterized
lpxC	UDP-3-O-acyl-N-acetylglucosamine deacetylase	Metabolism
mdtA_1	Multidrug resistance protein MdtA	Environmental Information Processing
ohrB	Organic hydroperoxide resistance protein OhrB	Unclassified: metabolism
oleC_2~oleC_1~ppsB	Olefin beta-lactone synthetase;Plipastatin synthase subunit B	Ligases

pilA_1	Fimbrial protein	Environmental Information Processing
pilA_2	Fimbrial protein	Environmental Information Processing
pilY1_5~pilY1_6~pilY1_1~ pilY1_3	Type IV pilus biogenesis factor PilY1	Protein families: signaling and cellular processes
ppsA_1~ppsA_2~ppsA	Phosphoenolpyruvate synthase	Metabolism
recG	ATP-dependent DNA helicase RecG	Genetic Information Processing
recQ	ATP-dependent DNA helicase RecQ	Genetic Information Processing
rpoA	DNA-directed RNA polymerase subunit alpha	Genetic Information Processing
secD_1~secD_2~secD	Protein translocase subunit SecD	Genetic Information Processing
sgcG	2-amino-4-deoxychorismate dehydrogenase	No KO assigned
spoT	Guanosine-3'5'-bis(diphosphate) 3'-pyrophosphohydrolase	Metabolism
sucC_1~sucC_2~sucC	Adenylosuccinate lyase	Poorly characterized
tatA	Sec-independent protein translocase protein TatA	Genetic Information Processing
tatC	Sec-independent protein translocase protein TatC	Genetic Information Processing
thrS	Threonine--tRNA ligase	Genetic Information Processing
tolB_2~tolB_1	Tol-Pal system protein TolB;Protein TolB	Protein families: signaling and cellular processes
ybaL	Putative cation/proton antiporter YbaL	Unclassified: signaling and cellular processes
yheS_2~yheS_1~yheS	putative ABC transporter ATP-binding protein YheS	genetic information processing

Table S3: Single nucleotide polymorphism GWAS results from Scoary, including loci with best pairwise comparison P < 0.05 and Bonferroni corrected P < 0.05. Annotations are also shown for available genes. 24 SNPs met these criteria, from 11 unique genes. Annotations of hypothetical proteins are abbreviated as HP. Scoary uses a phylogeny in order to remove lineage-specific interdependencies and offers both a multiple hypothesis corrected P value (Bonferroni P value) as well as a phylogenetically corrected P value (Best pairwise comparison P).

**Locus**	**Annotation**	**Number of significant SNPs in locus**	**Frequency in strains from *Vitis***	**Frequency in non-*Vitis* strains**	**Bonferroni p**	**Best pairwise Comp p**
aceE	Pyruvate dehydrogenase E1 component	1	0.45	0.14	5.74E-02	3.13E-02
envC	Murein hydrolase activator EnvC	1	0.45	0.01	7.22E-10	3.13E-02
fumC	Fumarate hydratase class II	7	0.44	0.03	7.62E-08	3.13E-02
gcvP_1~~~gcvP_2~~~gcvP	Glycine dehydrogenase (decarboxylating)	1	0.71	0.97	2.03E-02	3.13E-02
glnG	DNA-binding transcriptional regulator NtrC	2	0.46	0.01	5.64E-10	3.13E-02
group_1751	HU family DNA-binding protein	2	0.45	0.08	1.12E-04	3.13E-02
group_779	tail fiber domain containing protein	1	0.35	0.01	1.43E-05	3.13E-02
group_988	no results	2	0.07	0.54	3.62E-12	2.15E-02
uppP_1~~~uppP~~~uppP_2	Undecaprenyl-diphosphatase	1	0.46	0.03	1.07E-08	3.13E-02
xthA	Exodeoxyribonuclease III	3	0.45	0.14	5.74E-02	3.13E-02
yceI_3	Protein YceI	1	0.46	0.03	1.07E-08	3.13E-02

Table S4: Whole gene gain and loss GWAS results from Scoary, including loci with best pairwise comparison P ≤ 0.125 and Bonferroni P < 0.05. Annotations are also shown for available genes. Scoary uses a phylogeny in order to remove lineage-specific interdependencies and offers both a multiple hypothesis corrected P value (Bonferroni P value) as well as a phylogenetically corrected P value (Best pairwise comparison P).

**Locus**	**Annotation**	**Frequency in strains from *Vitis***	**Frequency in non-*Vitis* strains**	**Bonferroni p**	**Best pairwise Comp p**
group_1796	HP	0.84	0.50	3.09E-05	7.81E-03
group_1080	HP	0.92	0.71	5.12E-02	1.56E-02
group_1801	HP	0.66	0.08	1.22E-15	3.13E-02
group_1194	HP	0.53	0.04	1.74E-12	3.13E-02
group_1060	HP	0.93	0.69	2.07E-03	3.13E-02
xerC_1~~~xerC_2	Tyrosine recombinase XerC	0.69	0.93	3.50E-02	3.13E-02
group_605	HP	0.20	0.56	5.69E-05	3.86E-02
group_843	HP	0.26	0.74	1.93E-09	3.91E-02
group_787	HP	0.63	0.22	2.18E-06	3.91E-02
group_1349	HP	0.81	0.29	1.89E-12	6.25E-02
group_1320	HP	0.59	0.96	2.20E-07	6.25E-02
hcaB	3-phenylpropionate-dihydrodiol/cinnamic acid-dihydrodiol dehydrogenase	0.49	0.13	1.56E-05	6.25E-02
group_1046	HP	0.97	0.74	1.75E-05	6.25E-02
group_971	HP	0.93	0.65	5.88E-05	6.25E-02
group_1008	HP	0.94	0.74	8.46E-03	6.25E-02
group_661	HP	0.95	0.75	1.32E-02	6.25E-02
group_925	HP	0.95	0.75	1.32E-02	6.25E-02
group_451	HP	0.18	0.64	1.86E-09	7.03E-02
group_744	HP	0.18	0.49	1.47E-03	7.03E-02
group_817	HP	0.83	0.33	8.80E-12	1.09E-01

group_299	HP	0.11	0.53	3.10E-09	1.09E-01
group_1656	HP	0.85	0.47	1.29E-06	1.09E-01
group_846	HP	0.75	0.42	1.21E-03	1.09E-01
group_830	HP	0.60	0.28	3.45E-03	1.09E-01
group_504	HP	0.58	0.28	2.29E-02	1.09E-01
group_509	HP	0.81	0.50	2.21E-03	1.18E-01
group_1001	HP	0.19	0.69	1.84E-11	1.25E-01
group_809	HP	0.00	0.19	1.15E-06	1.25E-01
group_731	HP	1.00	0.81	1.15E-06	1.25E-01
group_1582	HP	0.49	0.11	5.17E-06	1.25E-01
mdtA_3~~~mdtA_2~~~mdtA_1	Multidrug resistance protein MdtA	0.49	0.13	3.11E-05	1.25E-01
cnrA~~~swrC_2~~~acrF_2	Nickel and cobalt resistance protein CnrA;Swarming motility protein SwrC;Multidrug export protein AcrF	0.49	0.13	3.11E-05	1.25E-01
group_181	HP	0.49	0.13	3.11E-05	1.25E-01
group_1750	HP	0.97	0.75	6.92E-05	1.25E-01
group_710	HP	0.02	0.22	9.77E-05	1.25E-01
group_201	HP	0.02	0.22	9.77E-05	1.25E-01
group_1188	HP	0.02	0.22	9.77E-05	1.25E-01

Figure S1: *Helianthus annuus* and *Vitis vinifera* positive controls. 4 Chardonnay 1 year old rooted cuttings were inoculated per treatment and 10 5-week-old sunflower plants per treatment. Mean C_q values of positive samples (C_q < 37) are labeled on each bar. ALS15-2 is the one subspecies *multiplex* strain used for the inoculations.

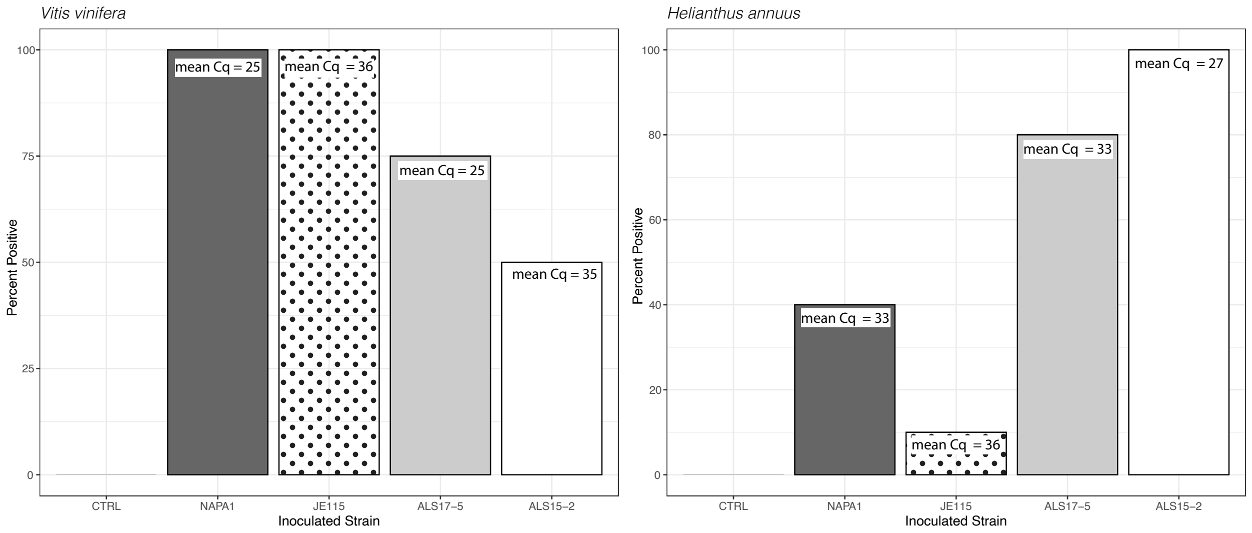
Vitis vinifera
Percent Positive
Inoculated Strain
CTRL
NAPA1
JE115
ALS17−5
ALS15−2
0
25
50
75
100
mean Cq = 25
mean Cq = 36
mean Cq = 25
mean Cq = 35
Helianthus annuus
Percent Positive
Inoculated Strain
CTRL
NAPA1
JE115
ALS17−5
ALS15−2
0
20
40
60
80
100
mean Cq = 33
mean Cq = 36
mean Cq = 33
mean Cq = 27

Figure S2: Mean C_q values from positive (C_q<37) *C. arabica* plants qPCR sampled in July and October, 2022. In July, one leaf from each plant was sampled 1 internode above the inoculation point, and in October one leaf from each plant was sampled 2 internodes above the inoculation point.

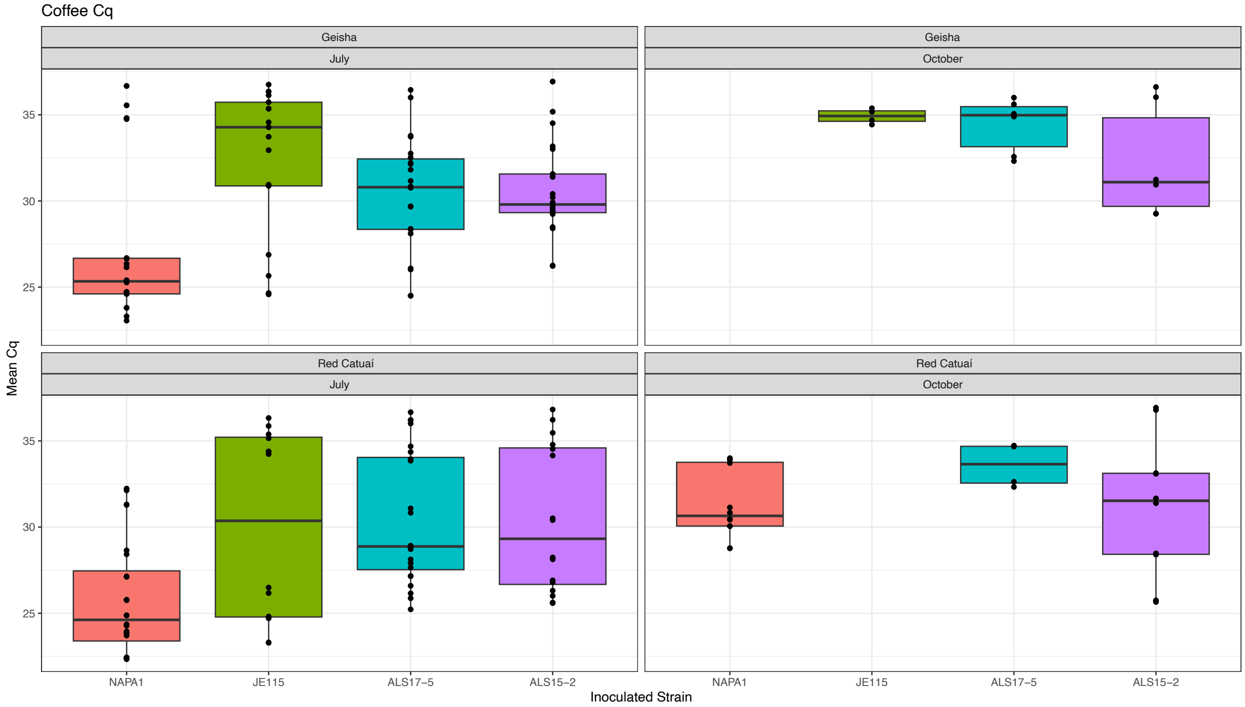

Figure S3: TreeWAS subsequent score distribution for all SNPs with location in genome noted for 6 SNPs significantly associated with the host *Vitis* spp.

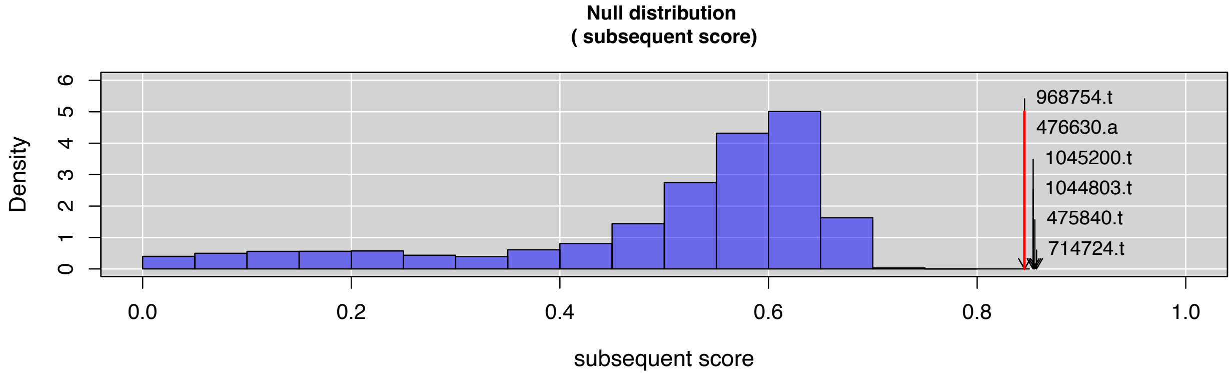

Figure S4: Gene trees of 5 loci (6 SNPs across 5 genes) identified using treeWAS (wbbL, pilT_2, pilT_1, group_86, and group_1468). Clades are labeled with an image of the most

frequent host plant strains were isolated from: Almond, Coffee, or Grape.

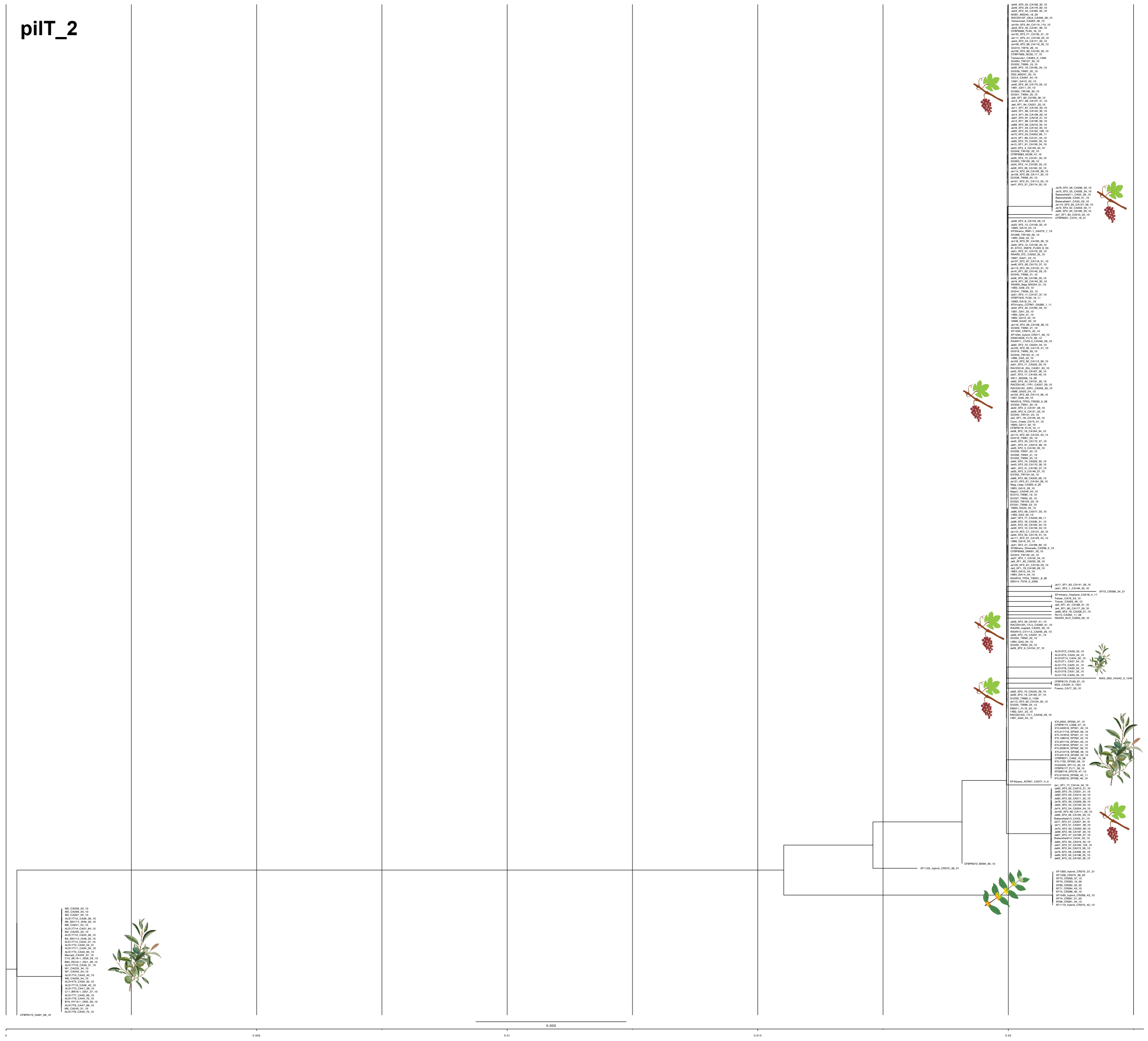

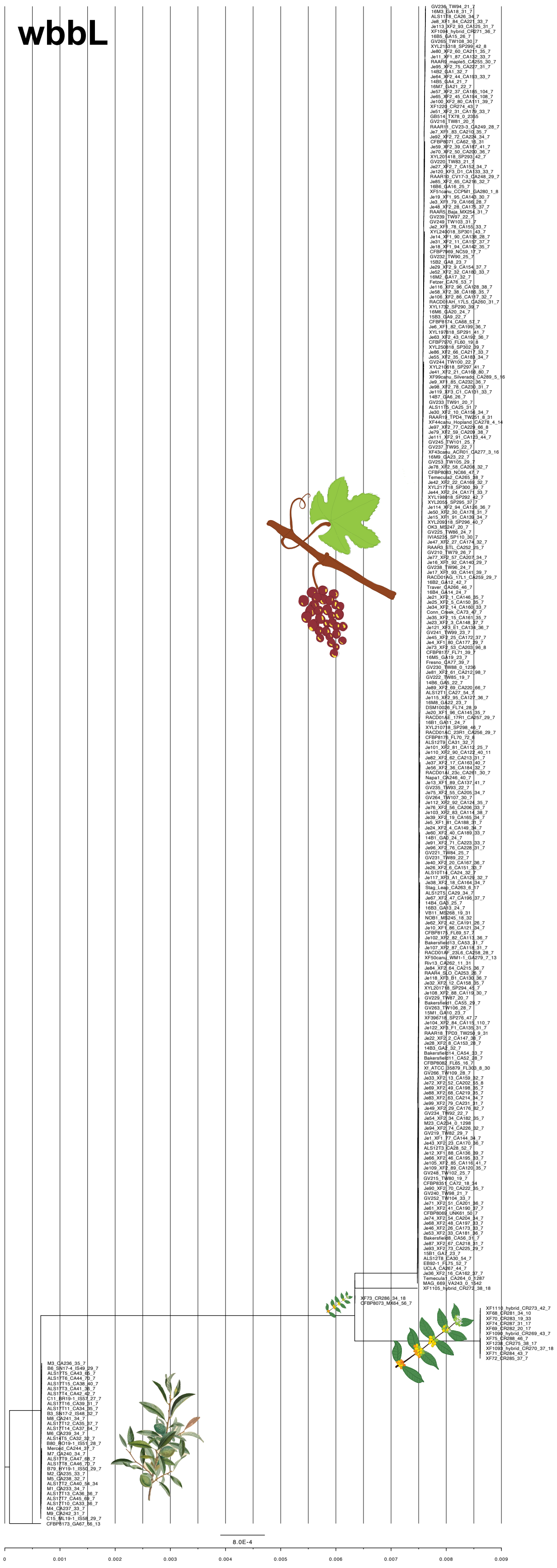
wbbL
8.0E-4
0
0.001
0.002
0.003
0.004
0.005
0.006
0.007
0.008
0.009

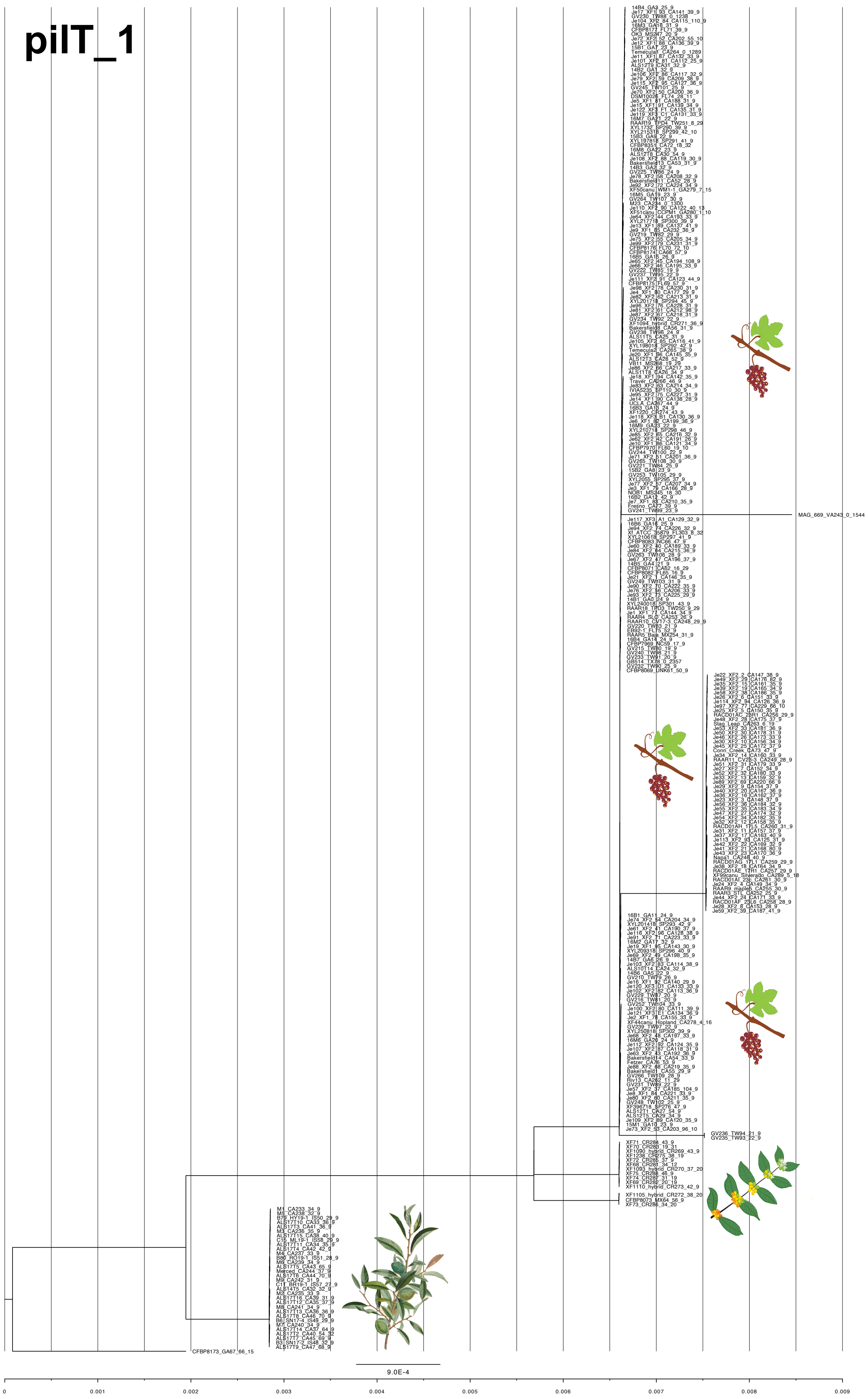
pilT_1
MAG_669_VA243_0_1544
CFBP8173_GA67_66_15
9.0E-4
0
0.001
0.002
0.003
0.004
0.005
0.006
0.007
0.008
0.009

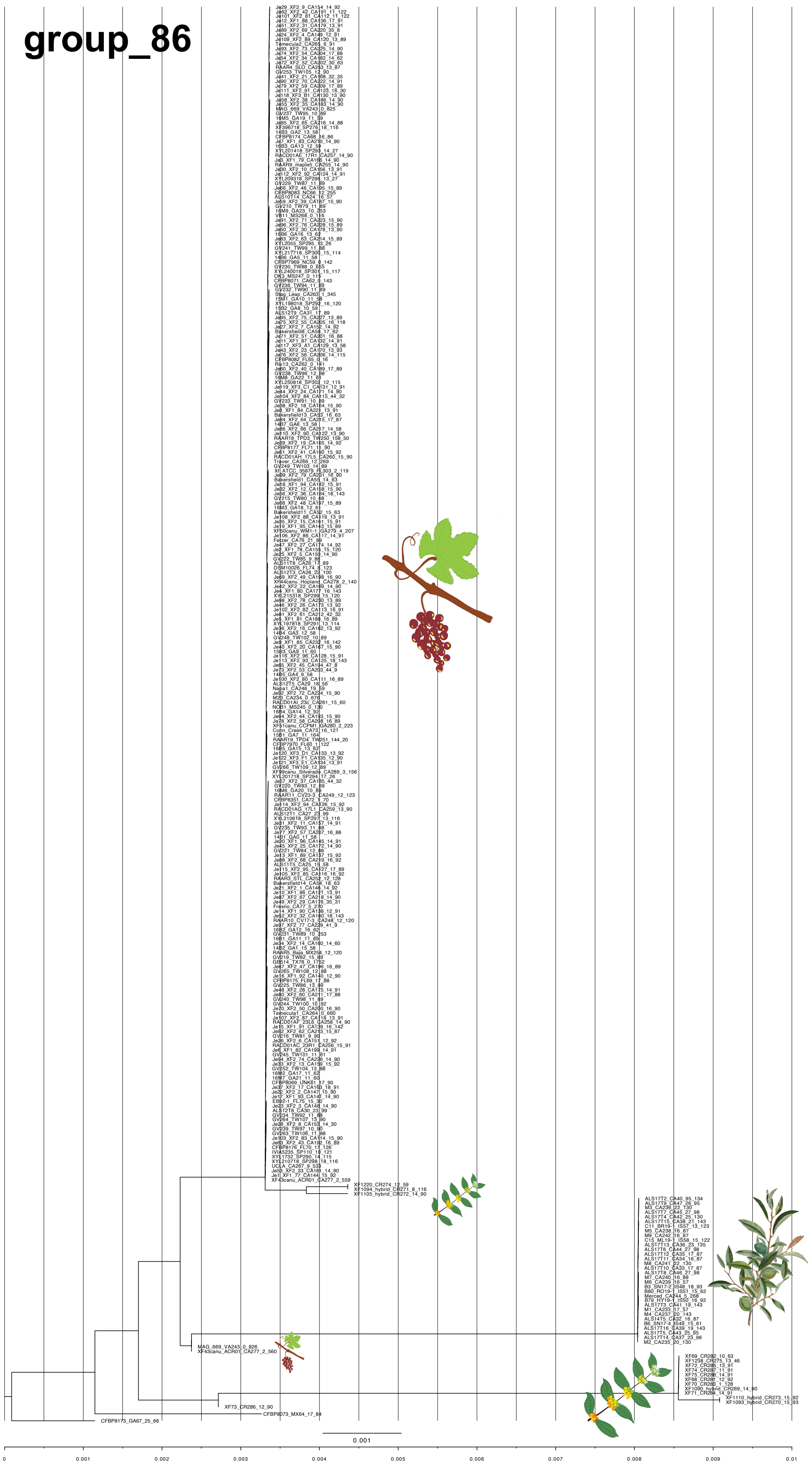
group_86
0.001
0
0.001
0.002
0.003
0.004
0.005
0.006
0.007
0.008
0.009
0.01

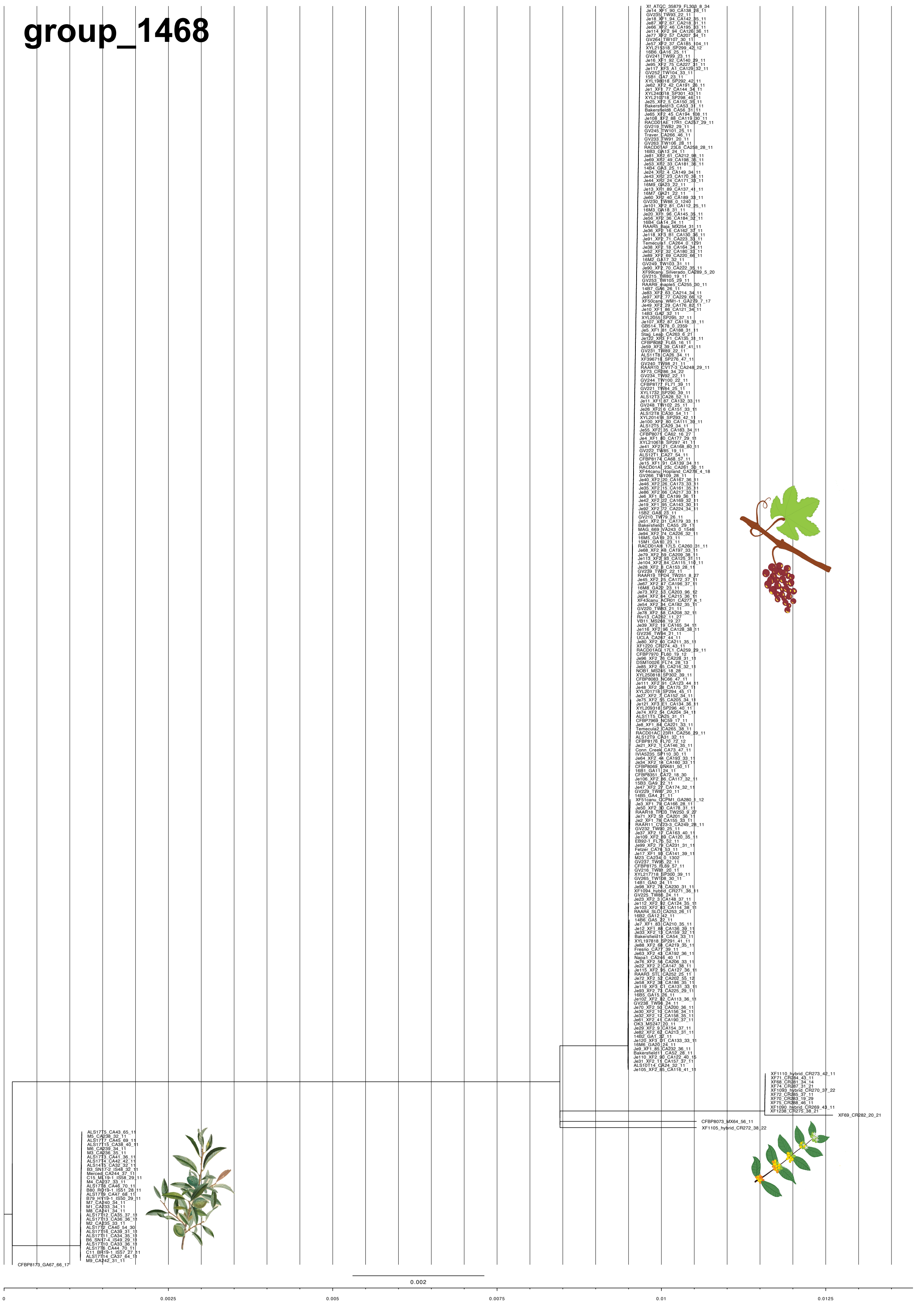
group_1468
XF69_CR282_20_21
CFBP8073_MX64_56_11
XF1105_hybrid_CR272_38_22
CFBP8173_GA67_66_17
0.002
0
0.0025
0.005
0.0075
0.01
0.0125
